# Review: Efficacy of preventative interventions for children and adolescents at clinical high risk of psychosis – a systematic review and meta‐analysis of intervention studies

**DOI:** 10.1111/camh.12755

**Published:** 2024-12-17

**Authors:** Grace Frearson, Javier de Otazu Olivares, Ana Catalan, Claudia Aymerich, Gonzalo Salazar de Pablo

**Affiliations:** ^1^ Department of Child and Adolescent Psychiatry Institute of Psychiatry, Psychology & Neuroscience, King's College London London UK; ^2^ School of Medicine Universidad Nacional Pedro Henríquez Ureña Santo Domingo Dominican Republic; ^3^ Early Psychosis: Interventions and Clinical‐detection (EPIC) Lab, Department of Psychosis Studies Institute of Psychiatry, Psychology & Neuroscience, King's College London London UK; ^4^ Psychiatry Department, Biocruces Bizkaia Health Research Institute, OSI Bilbao‐Basurto, Facultad de Medicina y Odontología University of the Basque Country UPV/EHU, Centro de Investigación en Red de Salud Mental (CIBERSAM), Instituto de Salud Carlos III Barakaldo Bizkaia Spain; ^5^ Child and Adolescent Mental Health Services (CAMHS) South London and Maudsley NHS Foundation Trust London UK; ^6^ Department of Child and Adolescent Psychiatry, Institute of Psychiatry and Mental Health, Hospital General Universitario Gregorio Marañón School of Medicine Universidad Complutense, Instituto de Investigación Sanitaria Gregorio Marañón (IiSGM), CIBERSAM Madrid Spain

**Keywords:** Clinical high risk, psychosis, interventions, children, adolescents

## Abstract

**Background:**

Despite evidence suggesting that age moderates the response to preventative treatment for those at clinical high risk of psychosis (CHR‐P), no meta‐analysis has assessed the effectiveness of preventative interventions for CHR‐P children and adolescents. Our aim was to synthesise evidence assessing preventative interventions on a wide range of mental health outcomes for CHR‐P children and adolescents.

**Method:**

A systematic search was conducted on Ovid MEDLINE, Pubmed, APA PsycInfo and Web of Science until June 2024 (PROSPERO: CRD42023406696). Intervention studies that had a mean participant age of under 18 years old that reported on mental health outcomes for CHR‐P participants were selected. A meta‐analysis was conducted for independent studies reporting the effectiveness of interventions on different outcomes (transition to psychosis, attenuated positive, negative and total prodromal psychotic symptoms, depressive symptoms and global functioning) compared to control conditions of no intervention or placebo. Evidence from other studies was also reported narratively.

**Results:**

Twenty‐four studies and 1319 CHR‐P children and adolescents were included. Compared to no intervention or placebo, preventative interventions were effective for positive symptoms (SMD = 0.379, *p* = .022, 95% CI 0.055, 0.703), negative symptoms (SMD = 0.583, *p* = .004, 95% CI 0.187, 0.980), total symptoms (SMD = 0.677, *p* = .002, 95% CI 0.249, 1.105) and functioning (SMD = 0.944, *p* = .038, 95% CI 0.052, 1.836) but not reducing transition to psychosis or depressive symptoms.

**Conclusions:**

There are disparities in the effectiveness of preventative interventions for different outcomes, with transition to psychosis not being the only relevant outcome. Differences in the efficacy of preventative interventions emerged between CHR‐P children and adolescents versus adults.

**Key Practitioner Messages**

**What is known?**

- The Clinical High‐Risk for Psychosis (CHR‐P) paradigm is relevant throughout different stages of development, including children and adolescents. There is some preliminary evidence that cognitive behavioural therapy can reduce some mental health outcomes (e.g. the risk of transition to psychosis) in adults and other pharmacological and psychotherapeutic interventions have been piloted for different mental health outcomes.

**What is new?**

- Preventative interventions for CHR‐P adolescents can improve attenuated positive psychotic symptoms, negative symptoms and total symptoms as well as functioning with small to moderate effect sizes. Currently available preventive interventions have not shown a reduction in transition to psychosis or depression symptoms in adolescents.

**What is significant for clinical practice?**

- Preventive interventions for CHR‐P adolescents have the potential to improve mental health outcomes other than transition to psychosis. Further research is required to recommend one intervention over others, with adverse effects and acceptability encompassed to find the most effective intervention with the best safety profile.

## Introduction

The World Health Organisation listed psychotic disorders as some of the most burdensome and costly illnesses, accounting of 12.2% of all mental health disorder disability adjusted life years (DALYs) (GBD, 2021). Given the significant impact of these disorders coupled with their tendency to manifest at an early age (Solmi et al., 2022), preventative interventions are paramount within this population.

The introduction of the Clinical High‐Risk for Psychosis (CHR‐P) paradigm over the last two decades has become one of the most established approaches for preventative intervention with increasing numbers of specialist services developed to meet the needs of CHR‐P individuals (Kotlicka‐Antczak et al., 2020). Individuals at CHR‐P are typically between 12 and 25 years old and accumulate risk factors for developing a psychotic disorder over the following 2–5 years.

Psychometric assessments including the Comprehensive Assessment of At‐Risk Mental State (CAARMS; Yung et al., 2005) or the Structured Interview of Psychosis‐Risk Syndromes (SIPS/SOPS; Miller et al., 1999) identify which individuals fulfil criteria for at least one of the three CHR‐P subgroups: attenuated psychotic symptoms (APS, representing 85% of cases) including perceptual disturbance, paranoia, unusual ideas or ideas of reference, brief, limited and intermittent psychotic symptoms (BLIPS, 10% of cases) who experience positive symptoms but with decreased duration and remitting without anti‐psychotic medication or genetic risk and deterioration syndrome (GRD, 5%) (Fusar‐Poli et al., 2013).

Given the transition rate of 25% to a first episode of psychosis within 3 years from the CHR‐P state (Salazar De Pablo et al., 2021) as well as the poor functioning and symptomology associated with the CHR‐P state (Salazar De Pablo et al., 2021), it is essential that, once identified, CHR‐P individuals receive effective care in order to improve long‐term outcomes and functionality.

As suggested in the Institute of Medicine's framework, preventative interventions include all actions taken before a specific diagnostic threshold is met. Within this framework, preventative interventions implemented for CHR‐P individuals are situated between indicated primary prevention (based on the premise that these individuals have a particularly high vulnerability to developing a psychotic disorder, along with prodromal symptoms) and secondary prevention (especially in cases where the transition to a first psychotic episode becomes inevitable) (Robinson, Haaz, Petrica, Hillsberg, & Kennedy, 2004).

Several meta‐analyses have evaluated the effectiveness of interventions for CHR‐P individuals, with transition to psychosis as the main outcome in adults. In general, preventative interventions were found to reduce the risk of transition to psychosis across 6–48 months follow up compared to controls (Mei et al., 2021; Schmidt et al., 2015), with one meta‐analysis reporting a 54% reduction in risk of transition compared to controls (Van Der Gaag et al., 2013). Other meta‐analyses have suggested that preventative interventions, when psychological and pharmacological interventions were pooled together, significantly reduced attenuated positive symptoms but did not reduce negative symptoms, depressive symptoms nor improve functioning (Mei et al., 2021; Schmidt et al., 2015; Stafford, Jackson, Mayo‐Wilson, Morrison, & Kendall, 2013). Network meta‐analyses also found no evidence to suggest that one preventative intervention was significantly better than others across multiple different mental health outcomes including the transition to psychosis risk (Davies et al., 2018), attenuated positive symptoms (Davies et al., 2018) and negative symptoms (Devoe, Peterson, & Addington, 2018).

However, all of these meta‐analyses focused on adult populations. Help‐seeking CHR‐P children and adolescents exhibit a risk of developing psychosis comparable to that of adult help‐seekers, confirming the relevance of the CHR‐P paradigm throughout different stages of development (Raballo, Poletti, Preti, & McGorry, 2022). Schmidt et al. (2015) meta‐analysis including adults suggested that preventative interventions were less effective at reducing transition to psychosis rate for younger participants although this was not significant. Furthermore, gains in functioning after preventative interventions were significantly larger for younger participants. Both of these findings suggest that age could moderate response to preventative interventions. Nonetheless, the effectiveness of preventative interventions among this specific population remains mostly unknown.

Based on these gaps in existing literature, this meta‐analysis presents evidence from preventative intervention studies on CHR‐P children and adolescents that include multiple mental health outcomes for assessing the effectiveness of the intervention.

## Methods

This review was carried out in accordance with the Preferred Reporting Items for Systematic Reviews and Meta‐Analyses (PRISMA; Moher et al., 2016; Tables S1 and S2) and the Meta‐analysis of Observational Studies in Epidemiology (MOOSE) checklist (Stroup et al., 2000; Table S3). The review's protocol was registered on PROSPERO: CRD42023406696.

### Search strategy and selection criteria

A systematic search on Ovid MEDLINE, PubMed, APA PsycInfo and Web of Science was conducted from inception until 20 June 2024 by two independent researchers (GF, JOO) using the following search terms: (‘child* or adolescen* or teen* or paediatric or youth’) AND (‘prodrom* or ultra high risk or clinical high risk or high risk or attenuat* or APS or brief limited or brief intermittent or BLIPS or genetic high risk or GRD or at risk mental states or risk of progression to first episode or basic symptoms’) AND (‘psychosis or psychotic disorder’) AND (‘treat* or interve* or psychological interve* or psychosocial interve* or pharmacolog* interve* or prevent* interve* or medicat*’).

A manual search was conducted to identify additional studies using backwards and forwards citation chasing. Additional searches were conducted on Cochrane CENTRAL and ProQuest dissertations and theses to search for grey literature using more limited search terms as part of the manual search: (‘clinical high risk of psychosis’ or ‘CHR‐P’) AND (‘children’ or ‘adolescent*’) AND (‘intervention’) AND (‘psychosis’ or ‘psychotic disorder’). The identified studies were first screened using their title and abstract, excluding studies that were not relevant. The remaining studies were then assessed for eligibility using the full text and applying the following inclusion criteria: (a) studies where the mean age of participants is under 18 in line with existing systematic reviews and meta‐analyses (Catalan et al., 2021), (b) participants identified to meet CHR‐P criteria using validated instruments (Methods S1), (c) studies that assessed mental health outcomes (definitions in Methods S2), (d) intervention studies with a longitudinal design assessing change in these mental health outcomes over time. Any studies meeting the following criteria were excluded: (a) case studies, reviews, conference abstracts or study protocols, (b) studies conducted on those with first‐episode psychosis or other populations (e.g. non‐CHR‐P), (c) studies that were not written in English. For the meta‐analysis, only intervention studies that included the control group of treatment as usual, no intervention or placebo were considered. Furthermore, at least two studies are needed to report on the same mental health outcome. Where there was any overlap in the samples used in the studies, the study with the larger and more representative sample was included in the meta‐analysis.

### Data extraction

Two independent researchers conducted the data extraction (GF and JOO) and a third independent researcher was consulted to resolve discrepancies (GSP). The variables extracted from each eligible study were: first author and publication year, study design, country, intervention and control group, components of psychological interventions, number and mean age of participants in intervention and control groups, percentage of males, instrument to assess CHR‐P, scales used to assess outcomes, length of intervention and to follow up assessments, change scores for each outcome and key findings. A detailed data extraction table can be seen in Table S4.

### Risk of bias assessment

This review used version 2 of the Cochrane Risk‐of‐Bias tool (ROB 2;Sterne et al., 2019) to assess the quality of the randomised control trials (RCT) and the quality of the eligible nonrandomised studies of intervention (NRSI) and naturalistic studies. Each study was assessed based on the randomisation process, deviations from intended interventions, missing outcome data, measurement of outcomes and selection of reported results. The Risk of Bias in Non‐Randomised Studies of Interventions tool (ROBINS‐I; Higgins et al., 2011) also assessed the quality of the eligible NRSIs and naturalistic studies.

### Assessment of the certainty of evidence

We assessed the certainty of evidence for each outcome using the GRADE approach (Guyatt et al., 2008). When we assessed the certainty of evidence for outcomes that only included data from RCTs, the certainty started at high quality and then was downgraded based on risk of bias, imprecision, indirectness, inconsistency of evidence and publication bias. The certainty was upgraded when there was a large effect, a clear dose–response or when any confounding was likely to decrease the magnitude of the effect. The certainty of evidence for outcomes that included data from observational studies started as low quality and was downgraded or upgraded based on the same criteria.

### Meta‐analysis strategy

We reported between‐group differences between preventative interventions and inactive control groups. The rationale behind this decision was based on Cochrane guidelines that suggest that all interventions and comparators included in a meta‐analysis should be similar enough to be combined meaningfully (Ryan, 2016). Whilst there were intervention studies that compared two active interventions and met the criteria for inclusion in this study, the active comparators across relevant studies were so different that they could not be combined meaningfully.

Due to the high‐expected heterogeneity, we used random effects modelling (Dersimonian & Laird, 1986). Six outcomes were considered for the meta‐analysis: transition to psychosis (primary outcome), attenuated positive symptoms, negative symptoms, total symptoms, depressive symptoms and functioning. Heterogeneity among study estimates was assessed with the Q statistic, with the proportion of the total variability in effect size estimates due to between‐study heterogeneity was evaluated using the I2 index (Lipsey & Wilson, 2001). To evaluate the efficacy of the preventative interventions on transition to psychosis, odds ratio was used as effect size. Standardised mean difference (SMD) (95% CI) was used as effect size for continuous outcomes. Too few studies reported the specific age range of the final samples used in their analysis to conduct a sensitivity analyses comparing the preventive effect on studies including some individuals over 18‐year‐old and those not including any individual 18 years or older. However, previous meta‐analysis that conducted this sensitivity analysis found no differences in preventative effect (Catalan et al., 2021).

Publication bias was assessed by conducting an Egger's test and funnel plots (Egger, Smith, Schneider, & Minder, 1997). To correct for missing studies when a risk of publication bias (e.g., a small sample size) was detected in the Egger's test, complementary ‘trim and fill’ method was used. The level of significance was *p* < .05 and all significance values reported were two‐sided. The meta‐analysis was conducted using Comprehensive Meta‐Analysis Software V3 (Borenstein & Higgins, 2013).

### Strategy for systematic review of studies unable to be included in meta‐analysis

Many of the eligible studies did not meet inclusion criteria for the meta‐analysis. Evidence from these studies is described in a narrative synthesis. Some of the eligible studies also reported outcomes such as family functioning, anxiety, disorganised symptoms, motor and cognitive deficits which were also not included in the meta‐analysis since the scales used to assess these outcomes and CHR‐P were too heterogeneous and few studies reported on the same outcome.

## Results

### Search results

**Figure 1:**
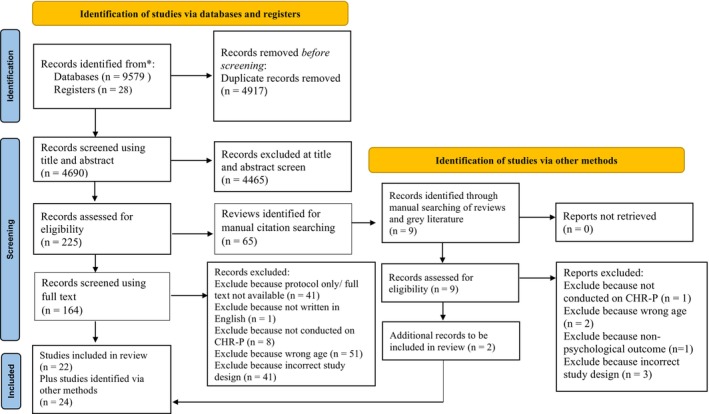
Adapted version of PRISMA 2020 flowchart detailing screening results

We identified 9607 relevant studies and, after removing duplicates, 4690studies were screened using their title and abstract. Of these studies, 161 underwent a full‐text screening process and 24 studies were finally included. Full details of the screening results are reported in an adapted version of the PRISMA 2020 flow diagram (Figure 1).

### Study characteristics

Twenty‐four studies including a total of 1319 CHR‐P participants with the mean age range from 14.4 (Pitzianti et al., 2019) to 17.6 years old (Brazzale, Maddalena, Cozzi, & Brazzale, 2018) were included in this review. 83.3% of the included studies did not provide the specific age range for the final samples used in the analyses. 51.6% of the included participants were male. Sample sizes ranged from *n* = 8 (Woods et al., 2013) to *n* = 292 (McFarlane et al., 2015). There were 13 RCTs (54.17%), 6 NRSIs (25%) and 5 naturalistic studies (20.83%). Ten studies assessed pharmacological interventions (41.67%) and 15 studies assessed psychological interventions (62.5%). The most common instruments to assess CHR‐P was the Structured Interview of Psychosis‐risk Syndromes/Scale of Prodromal Symptoms (SIPS/SOPS) (70.83%). The characteristics of the eligible 24 studies are summarised in Table 1, however, one study (Woods et al., 2013) reported the results of two separate trials.

**Table 1:** Study characteristics

Study number, authors and year	Study design	Intervention and control	Number of participants, mean age (SD) and age range^a^	Instrument to assess CHR‐P	Length of intervention; follow‐ups	Scales used to assess outcomes	Key findings	Risk of bias (ROB2): Positive symptoms^b^
1. Addington et al. (2023)	RCT	Cognitive behavioural social skills training (CBSST) vs. supportive therapy (ST).	CBSST: *n* = 70, age = 17.36 (4.01) ST: *n* = 82, age = 17.49 (4.12) Range: 12–30	SIPS	18 weeks; 18 weeks, 12 months	SOPS CDSS GF: R and GF: S	Regardless of therapy group, there were significant improvements in positive (*p* < .001), negative symptoms (*p* < .05) and depression symptoms (*p* < .5) at both 18 weeks as well as 12‐month follow‐up. Significant improvement in social functioning in the CBSST group (*p* < .01). There were no significant between‐group effects of CBSST versus ST in mean days to transition to psychosis, reduction in positive symptoms, reduction in negative symptoms, improvement in social or role functioning or reduction in depression symptoms at either 18 weeks or 12 months post‐intervention.	Low
2. Amminger et al. (2010)	RCT	Omega‐3 PUFA vs. coconut oil placebo	Omega‐3: *n* = 41, age = 16.8 (2.4) Placebo: *n* = 40, age = 16.0 (1.7) Range: 13–25	PANSS	3 months; 3, 6, 12 months	PANSS MADRS GAF	At 12 months, the intervention reduced risk of transition to psychosis compared to the control group (*p* = .007). The difference between intervention group and placebo in change from baseline to 12 months was significant for total symptoms (*p* = .006, *d* = 0.70), positive symptoms (*p* = .01 *d* = 0.69), negative symptoms (*p* = .02 *d* = 0.52) and functioning (*p* = .002 *d* = −0.72).	Low
3. Amminger et al. (2013)	RCT	Omega‐3 PUFA for CHR‐P with BPD vs. coconut oil placebo	Omega‐3: *n* = 8 Placebo: *n* = 7 Age for whole group: 16.2 (2.1) Range: 13–25	PANSS	12 weeks; 12 weeks	PANSS MADRS GAF	Significant improvements across time for intervention group in negative symptoms (*p* = .02, *d* = 0.92) and BPD symptoms (*p* = .01, *d* = 0.99). Within the PANSS BPD subscale, significant change in depressive symptoms (*p* = .005), suspiciousness (*p* = .009), emotion withdrawal (*p* = .02) and anxiety (*p* = .03). Significant change in functioning from baseline to after intervention (*p* = .01, *d* = 1.59)	Low
4. Amminger, Schäfer, Schlögelhofer, Klier, and McGorry (2015)	RCT follow up	Omega‐3 PUFA vs. coconut oil placebo	Omega‐3: *n* = 41, age = 16.8 (2.4) Placebo: *n* = 40, age = 16.0 (1.7) Range: 13–25	PANSS	3 months; median of 6.7 years	PANSS MADRS GAF	Significant difference between treatment groups in conversion to psychosis (95% CI 10.1–50.4). Omega‐3 group had significantly lower PANSS scores (positive, negative and total symptoms, *p* < .05 for all), higher functioning (*p* = .011) and lower depression (*p* = .021) at longer term follow up than placebo.	Low
5. Bowie, Mclaughlin, Carrion, Auther, and Cornblatt (2012)	NRSI	Antidepressant and antipsychotic medication vs. off medication and HC	Antidepressants: *n* = 15, age = 15.5 (1.9) Antipsychotics: *n* = 11, age = 16.4 (1.5) Off meds: *n* = 27, age = 16.4 (2.0) HC: *n* = 17, age = 16.4 (2.3) Range: 12–22	SIPS/SOPS	Treated for at least 3 months before follow up; 6 months	SIPS BDI BAI Cognitive deficits	Effect of time for positive symptoms (*p* = .02) and depressive symptoms (*p* = .004) but no group differences (*p* = .97 and *p* = .88, respectively). Greater improvement in verbal learning for antidepressants group (*p* = .027, *d* = 0.63) over antipsychotics group (*d* = −0.33, performance got worse). Greater improvement in sustained attention (CPT) for antidepressant group (*p* = .003, *d* = 1.02) than in OFF meds (*d* = 0.80) and greater improvement in OFF meds group compared to antipsychotics (*d* = −0.39, performance got worse).	High
6. Brazzale et al. (2018)	Naturalistic	Video therapy with metacognitive techniques (no control group)	*n* = 18, age = 17.6 (3.0) Range: 12–21	ERIraos Checklist	4 weeks; 4 weeks	YSR Cognitive deficits	Significant changes between pre intervention and post intervention in YSR anxious/depressed (*p* < .01), YSR total problems score (*p* < .01). Improvement that approached significance between baseline and after intervention in ability to interpret and analyse social scenarios (*p* = .06).	For outcome of YSR: High
7. Cannon et al. (2022)	RCT	FFT for CHR‐P with and without comorbid anxiety disorder vs. EC (family psychoeducation)	Anxiety FFT: *n* = 32 Nonanxiety FFT: *n* = 34 Anxiety EC: *n* = 35 Nonanxiety EC: *n* = 28 Anxiety group age = 17.1 (3.7) Nonanxiety group age = 17.6 (4.4) Range: 12–35	SIPS	Treated for at least 8 weeks; every 6 months for up to 5 years	Zung SAS Family communication	Anxiety decreased more in FFT than in the EC from pre‐treatment to 12 month follow up (*p* = .02, η^2^_*p*_ = .116) but no significant differences between treatment groups at 6 months. Those in the EC reported an increase in anxiety symptoms at 12 months compared to a further reduction in anxiety symptoms in the FFT group from the 6 month mark.	For outcome of anxiety: Some concerns
8. Cornblatt et al. (2007)	Naturalistic	Antidepressants vs. second generation antipsychotics	Antidepressants: *n* = 20, age = 16.3 (2.6) Antipsychotics: *n* = 28, age = 15.7 (1.9) Range: 12–22	SIPS/SOPS	12 months; 12 months	SIPS Medication adherence	Antidepressants reduced transition to psychosis risk more than antipsychotics (*p* = .007). three out of five positive symptoms (suspiciousness, unusual thoughts and perceptual abnormalities) improved with both medications across time (*p* < .001 for all three symptoms). Eleven of the 12 participants who transitioned to psychosis were nonadherent to medication and this difference in conversion to psychosis between levels of adherence was significant (*p* = .001). Participants were more likely to be nonadherent to antipsychotics than to antidepressants (*p* = .005).	High
9. Grano et al. (2016)	NRSI	FCTM vs. TAU	FCTM: *n* = 28, age = 15.5 TAU: *n* = 28, age = 16.3 (0.8) Range: 12–22	PROD‐screen then SIPS/SOPS	8 weeks; 9th week	SIPS BDI BAI GAF	No significant difference between treatment groups in prodromal symptoms (*p* = .727, *d* = −0.05) or anxiety (*p* = .059, *d* = −0.52). Significant difference between treatment groups in favour of FCTM in improvement in GAF (*p* = .004, *d* = 0.68), reduction in depression (*p* = .012, *d* = −0.76).	High
10. Holzer et al. (2014) (first published 2012)	RCT	CACR vs. computer games	CACR: *n* = 18, age = 15.4 (1.3) Computer games: *n* = 14, age = 15.7 (1.4) Range: N/A	SIPS/SOPS	5 months; 5 months	PANSS SOFAS and hoNOSCA RBANS	There was a significant effect of time from baseline to week 9 such that positive and negative symptoms decreased (*p* < .01), functioning improved (*p* < .05), RBANS total improved (*p* < .01), memory improved (*p* < .05), attention improved (*p* < .01) and general psychopathology improved (*p* < .05). There were significant treatment group differences for change in visuospatial and constructional abilities (*p* < .05, *d* = 0.62).	High
11. Janssen, Maat, Slot, and Scheepers (2021)	Naturalistic	CBT alone and with PMT and FT for HR‐P vs. for HSP	For UHR‐P: *n* = 61, age = 15.8 (3.2) For HSP: *n* = 82, age = 17.1 (3.2) Range: 12–25	CAARMS	4 weeks; 4 and 8 weeks	CAARMS GAF and HoNOSCA	Reduction in APS in the CHR group (*p* < .001, *d* = 0.42). Improvement in functioning across time for both groups, GAF: *p* < .001, η^2^_*p*_ = .347, HNOSCA: *p* < .001, η^2^_*p*_ = .380, no group differences. Duration of treatment was an important determinant of functioning outcomes in both CHR and HSP groups (*p* < .01, η^2^_*p*_ = .08) but treatment type was not a significant determinant such that CBT was equally as effective as CBT plus add ons at changing functioning.	High
12. McAusland and Addington (2018)	Naturalistic	Heartrate variability biofeedback (no control group)	*n* = 20, age‐16.7 (2.3) Range: 13–22	COPS based on SIPS/SOPS	6 months; 6, 12, 24 months	COPS Zung SAS GF: R and GF: S	Unusual thought content (*p* < .01), grandiose ideas (*p* < .05) and overall positive symptoms (*p* < .05) significantly decreased from baseline to week 4 (*p* < .01) and at follow up. Significant reduction in dysphoric mood at week 4 (*p* < .001) and at follow up. There was a trend for improvement in anxiety that approached significance (*p* = .07).	High
13. McFarlane et al., 2015	NRSI	FACT for CHR‐P vs. community care	CHR‐P: *n* = 205, age = 16.4 (3.3) Community care: *n* = 87, age = 16.2 (3.2) Range: 12–25	SIPS/SOPS	6 months; 6 months	POPS SIPS GAF, GF: R and GF: S	Reduction of positive symptoms was greater for FACT group for CHR‐P compared to CLR comparison group. FACT superior to the control group for negative symptoms but only approached significance for CHR‐P (CHR: β = −1.90, *p* = .099). Improvements in GAF at 24 months were significantly larger for FACT(CHR‐P: Δ = 17.47, *p* < .001). Role (*p* = .03) and social (*p* < .0001) functioning both improved across time but no group differences. Degree of change was greater for EFEP than for CHR‐P.	High
14. Miklowitz et al. (2014)	RCT	FFT vs. EC (family psychoeducation)	FFT: *n* = 66, age = 17.3 (4.2) EC: *n* = 63, age = 17.4 (3.9) Range: 12–32	SIPS/SOPS	3 months; 3, 6, 12 months	SIPS GAF, GF: R and GF: S	Being in EC group rather than the FFT group increased risk of transition (OR = 4.7). Significant effect of time (*p* < .0001) but no effect of treatment group for change in total SOPS symptoms, negative symptoms or GAF. Greater improvement from baseline to 6 months in FFT compared to EC for positive symptoms (*p* = .02, *d* = 0.56). For older adolescents (16–19) EC was associated with a greater improvement in GAF and role functioning than FFT (*p* < .05, *d* = −0.67 and *p* = .004, *d* = −0.94, respectively).	High
15. Mossaheb et al. (2013)	RCT follow‐up	Omega‐3 PUFA vs. coconut oil placebo	Omega‐3: *n* = 41, age = 16.8 (2.4) Placebo: *n* = 40, age = 16.0 (1.7) Range: 13–25	PANSS	6 months; 6 months	PANSS MADRS GAF	Omega 3 intervention's significant effects on total PANSS scores are clear after first 4 weeks of treatment, a reduction of positive symptoms and a lower mean PANSS positive score is apparent after 8 weeks. This is compared to the drop in negative symptoms and improvement in GAF occurring later at 12 weeks.	Low
16. O'Brien et al. (2007)	Naturalistic	PMFG vs. declined participation	PMFG: *n* = 16, age = 15.7 Declined participation: *n* = 13, age = 16.1 Range: 12–22	SIPS/SOPS	9 months; 9 months	SIPS GAF Family adaptability	No difference between groups in conversion to psychosis. PMFG group showed a significant reduction in positive symptoms (*p* < .01) and a reduction in general prodromal symptoms (*p* < .01) between baseline to follow‐up. Improvement in GAF (*p* < .05), occupational functioning (*p* < .05), family cohesion and adaptability (*p* < .01 for both) as well as an increase in constructive coping (*p* < .05) from baseline to follow‐up.	High
17. O'Brien et al. (2014)	RCT follow‐up	FFT vs. EC (family psychoeducation)	FFT: *n* = 38, age = 17.2 (4.2) EC: *n* = 28, age = 16.5 (2.5) Range: 12–35	SIPS/SOPS	6 months; 6, 12 months	Family interaction (family behaviour observational assessment)	FFT group expressed higher levels of constructive behaviour than EC at 6 months (*p* = .004). Those in FFT showed a significant increase in calm‐constructive (*p* < .0001) and a significant decrease in critical‐conflictual behaviour (*p* < .0001).	For outcome of family behaviour: Low
18. Pitzianti et al. (2019)	Naturalistic	Risperidone vs. drug naïve and HC	Risperidone: *n* = 15, age = 15.2 (1.7) Drug naïve: *n* = 15, age = 14.6 (2.2) HC: *n* = 25, age = 14.4 (3.4) Range: 7–18	SIPS/SOPS	6 months; 6 months	PANESS	Significant differences between CHR‐P patients and healthy controls in overflow movements (*p* = .001), dysrhythmia (*p* = .001) and speed of timed activities (*p* = .001). No significant differences between CHR‐P risperidone and drug naïve group in overflow movements (*p* = .886), dysrhythmia (*p* = .208) or speed of timed activities (*p* = .822).	For outcome of PANESS: High
19. Stain et al. (2016)	RCT	CBT vs. NDRL	CBT: *n* = 30, age = 16.2 (2.7) NDRL: *n* = 27, age = 16.5 (3.2) Range: 14–30	CAARMS	6 months; 6, 12 months	CAARMS SIPS BSI GAF	Transition rate was 5% with all three transitions in the CBT group and none in the NDRL group. Treatment effect in favour of NDRL for distress related to prodromal symptoms (treatment effect = 36.71, *p* = .029, 95% CI 3.71–69.71).	Some concerns
20. Urben, Pihet, Jaugey, Halfon, and Holzer (2012)	RCT	CACR vs. computer games for CHR‐P and diagnosed psychotic disorders	CACR: *n* = 12, 16.6% CHR‐P, age = 15.2 (1.3) Computer games: *n* = 10, 40% CHR‐P, age = 16.0 (1.3) Range: N/A	SIPS/SOPS	8 weeks; 6 months	Processing speed, working memory, executive functioning (EF) and reasoning.	Significant improvement in executive functioning in CACR group between baseline and follow‐up (*p* = .04, partial eta squared = 0.348) and a significant improvement in reasoning and planning for CACR (*p* = .005, partial eta squared = 0.621) but not for control group. Significant reduction in symptom severity from baseline to follow‐up for the control group (*p* = .046, partial eta squared = 0.400) but only a marginal decrease in symptom severity in the CACR group that approached significance (*p* = .088).	For outcome of EF: High
21. Waite et al. (2023)	RCT	SleepWell therapy vs. usual care (UC)	Sleepwell: *n* = 21, age = 17.0 (2.2), UC: *n* = 19, age = 16.8 (2.8) Range: 14–23	CAARMS	12 weeks; 3 months, 9 months	CAARMS R‐GPTS SPEQ‐H DASS‐21 W&SAS	One person in the UC group transitioned to psychosis. At 3 months after the interventions, there was a favourable effect of Sleepwell over usual care for psychotic experiences measured by CAARMS, depression and paranoid thoughts but none of these were significant between‐group differences. At 9 months after the intervention, there was a favourable effect of Sleepwell over usual care for psychotic experiences measured by CAARMS, depression and paranoid thoughts. These between‐group differences were significant for reduction in depression (*d* = 0.67), paranoid thoughts (*d* = 0.65). There was not a significant effect of Sleepwell over usual care for reducing total symptoms.	For outcome of total symptoms: Some concerns
22. Woodberry et al. (2021)	Naturalistic	Biofeedback computer‐aided learning for managing stress (no control group)	*n* = 11, age = 17.2 (2.3) Range: 12–30	COPS based on SIPS/SOPS	12 weeks; 12 weeks	SIPS GAF, GF: R and GF: S	No change over time in positive symptoms suggested to be because participants began this intervention after other treatments had stabilised their symptoms. Significant change between baseline and final assessment for social functioning (Hedge's *g* = 0.29, 95% CI 0.01–0.57).	High
23. Woods et al. (2007)	NRSI	Aripiprazole (no control group)	*n* = 14, age = 17.1 (5.5) Range: 13–40	COPS, SIPS/SOPS	8 weeks; 8 weeks	SIPS CDRS BAI GAF, GF: R and GF: S Cognitive deficits	Significant reduction for total SOPS score between baseline and 8 weeks (*p* < .001) and this reduction in SOPS was significant at each time point 1–8 weeks from baseline. Significant reduction in positive, negative, disorganisation and general SOPS scores (all *p* < .001). Significant reduction depressive symptoms (*p* < .005), anxiety (*p* < .001), improvement in GAF (*p* < .001), improvement in role functioning (*p* = .044). Significant worsening performance from baseline in attention and working memory (*p* = .040), but improvement in one task of letter‐number sequencing (*p* = .044) and significant reduction in semantic fluency (*p* = .008). There was a significant change in weight from baseline (*p* = .049).	High
24a. Woods et al. (2013)	RCT	Glycine vs. sucrose placebo	Glycine: *n* = 4, age = 15.3 (0.5) Placebo: *n* = 4, age = 16.5 (2.4) Range: 14–35	COPS, SIPS/SOPS	8 weeks; 8 weeks	SIPS MADRS GAF	No significant change in the 8 weeks for either control group or glycine groups for any sops measures or depression. Significant between groups difference in change in MADRS *p* < .05, *d* = −2.06 but no significant differences in change between treatment groups for any SOPS scores.	Some concerns
24b. Woods et al. (2013)	NRSI	Glycine (no control group)	*n* = 10, age = 17.3 (3.3) Range: 14–35	COPS, SIPS/SOPS	8 weeks; 8 weeks	SIPS MADRS GAF	Significant reduction in SOPS total at 8 weeks (SOPS total *d* = −1.39, SOPS positive *d* = −1.10), SOPS negative *d* = −0.74, SOPS disorganised *d* = −1.05 and in depressive symptoms (*d* = −0.44), all at *p* < .05.	High

Study design column: RCT, Randomised Controlled Trials; NRSI, Non‐Randomised Studies of Intervention. Intervention and control column: FFT, Family Focused Therapy; EC, Enhanced Care; FCTM, Family and Community Oriented Integrative Treatment Model; TAU, Treatment as Usual; FACT, Family‐Aided Assertive Community Treatment; EFEP, Early First Episode Psychosis; PMFG, Psycho‐educational Multi‐Family Group; CACR, Computer‐Aided Cognitive Remediation; PMT, Psychomotor Therapy; FT, Family Therapy; HSP, Help‐Seeking Population; NDRL, Non‐Directive Reflective Listening; BPD, Borderline Personality Disorder; PUFA, Polyunsaturated Fatty Acid; HC, Healthy Controls. Instrument to assess CHR‐P column: SIPS, Structured Interview for Psychosis‐Risk Syndromes; PROD, Screen for Prodromal Symptoms of Psychosis; SOPS, Scale of Prodromal Symptoms; COPS, Criteria of Prodromal Symptoms; CAARMS, Comprehensive Assessment of At‐Risk Mental States; ERIraos, Early Recognition Inventory for the Retrospective Assessment of Onset of Schizophrenia; PANSS, Positive And Negative Syndrome Scale. Instruments used to assess transition to psychosis: SIPS/SOPS, Structured Interview/Observation for Psychosis‐Risk Syndromes; PANSS, Positive and Negative Syndrome Scale; COPS, COPS, Criteria of Prodromal Symptoms; CAARMS, Comprehensive Assessment of At Risk Mental States; POPS, Presence of Psychosis Scale. Instruments to assess positive, negative and total psychotic symptoms: CAARMS, Comprehensive Assessment of At‐Risk Mental States; PANSS, Positive And Negative Syndrome Scale; SIPS/SOPS, Structured Interview/Observation for Psychosis‐ Risk Syndromes; R‐GPTS, Revised‐ Green Paranoid Thoughts Scale; SPEQ‐H, Specific Psychotic Experiences Questionnaire‐ Hallucinations. Instruments to assess depression and anxiety: MADRS, Montgomery‐Asberg Depression Rating Scale; BDI, Beck Depression Inventory; CDRS, Calgary Depression Rating Scale; BAI, Beck Anxiety Inventory; DASS‐21, Depression Anxiety Stress Scale 21 item; BSI, Basic Symptom Inventory; YSR, Youth Self Report; Zung SAS, Zung Social Anxiety Scale. Instruments sued to assess functioning: GAF, Global Functioning Scale; GF: R and GF: S, Role and social functioning; W&SAS, Work and Social Adjustment Scale; SOFAS, Social and Occupational Functioning Assessment Scale; HoNOSCA, Health of the National Outcome Scale for Children and Adolescents. Instruments to assess cognition: PANESS, Physical and Neurological Examination for Soft Signs; RBANS, Repeated Battery for Assessment of Neurological Status.

^a^ When available, reported age ranges are those included in the inclusion criteria when recruiting participants in each study as very few studies reported the final age range of the final sample.

^b^ Positive symptoms were the chosen outcome to demonstrate ROB2 assessment in this table as the ROB2 is outcome specific and the majority of studies included measurement of positive symptoms as an outcome. For those studies that did not measure positive symptoms, the risk of bias in their primary outcome is described in this column.

### Risk of bias

As seen in Table 1, the ROB2assessment found that 6 studies had a low risk of bias (24%), 4 had some concerns (16%) and 15 had a high risk of bias (60%). Most studies included in this systematic review are nonrandomised intervention studies. When assessed with the ROB2 tool, all these studies were deemed to have high risk of bias due to their lack of randomisation. Risk of bias assessments of the NRSIs and naturalistic studies using the ROBINS‐I can be found in the supporting information. Further information about the quality of each study in different domains can be seen in Figure S1 and Table S5.

### Meta‐analysis results

#### Transition to psychosis

**Figure 2:**
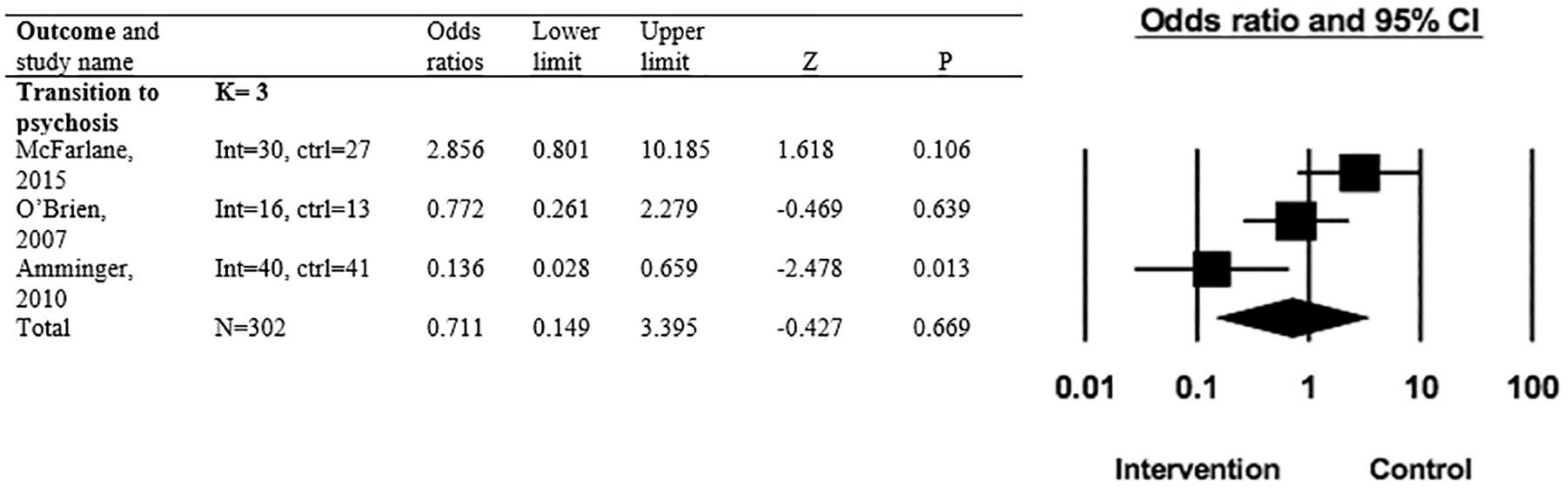
Forest plot for primary outcome of transition to psychosis

Three studies were included in the meta‐analysis for the outcome of transition to psychosis (Amminger et al., 2010; McFarlane et al., 2015; O'Brien et al., 2007). Overall, preventative interventions (including omega‐3 polyunsaturated fatty acid [PUFA], psychoeducational multi‐family groups [PMFG] and family aided assertive community treatment [FACT]) were not effective at significantly reducing transition to psychosis risk (*k* = 3, *n* = 402, OR = 0.711, 95% CI = 0.149–3.395, *p* = .669) (Figure 2). When included in the meta‐analysis, Omega‐3 PUFA was the only individual preventative intervention that reduced transition to psychosis risk significantly more than the control (OR = 0.14, *p* = .013). Heterogeneity across the included studies for transition to psychosis outcome was high (*Q* = 8.694, *I* ^2^ = 76.996, *p* = .013) (Deeks, Higgins, & Altman, 2023). As the evidence for this outcome came from RCTs and one observational study, the certainty of evidence started as moderate due to indirectness and then was further downgraded to low due to the results being analysed using a random effects model as heterogeneity was high.

### Attenuated positive psychotic symptoms

**Figure 3:**
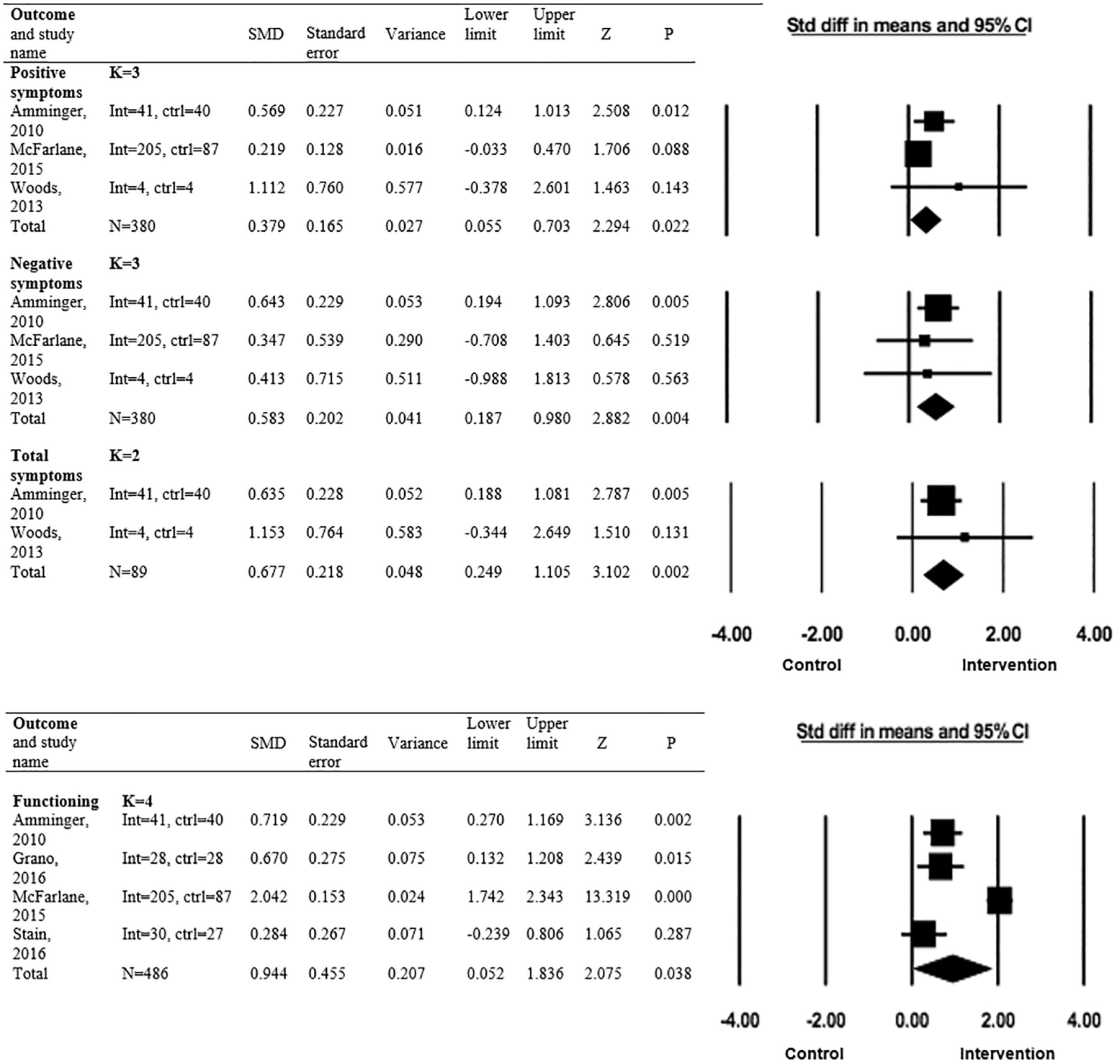
Forest plot for outcomes with significant total effects of preventative interventions over controls

Three studies were included in the meta‐analysis for the outcome of positive symptoms (Amminger et al., 2010; McFarlane et al., 2015; Woods et al., 2013). Overall, preventative interventions (including omega‐3 PUFA, FACT and glycine) were significantly more effective than controls at reducing positive symptoms (*k* = 3, *n* = 380, SMD = 0.379, 95% CI = 0.055–0.703, *p* = .022) (Figure 3). When included in the meta‐analysis, omega‐3 PUFA was the only individual intervention that reduced positive symptoms significantly more than control (SMD = 0.57, *p* = .012). Heterogeneity across the included studies was not significant (*Q* = 2.910, *I* ^2^ = 31.266, *p* = .233) but indicated there may be moderate heterogeneity according to Cochrane ranges (Higgins et al., 2024). The certainty of evidence was rated as moderate due to indirectness.

### Negative psychotic symptoms

Three studies were included in the meta‐analysis for the outcome of negative symptoms (Amminger et al., 2010; McFarlane et al., 2015; Woods et al., 2013). Overall, preventative interventions (including omega‐3 PUFA, FACT and glycine) were significantly more effective at reducing negative symptoms than controls (*k* = 3, *n* = 380, SMD = 0.583, 95% CI = 0.187–0.980, *p* = .004) (Figure 3). Again, when included in the meta‐analysis, omega‐3 PUFA was the only intervention that reduced negative symptoms significantly more than control (SMD = 0.64, *p* = .005). The heterogeneity across the studies was not significant (*Q* = 0.318, *I* ^2^ = 0.000, *p* = .853) and was unlikely to be important (Higgins et al., 2024) As the evidence for this outcome came from RCTs and one observational study, the certainty of evidence was rated as moderate due to indirectness.

### Total symptoms

Two studies were included in the meta‐analysis for the outcome of total symptoms (Amminger et al., 2010; Woods et al., 2013). Preventative interventions (including omega‐3 PUFA and glycine) were significantly more effective at reducing total prodromal symptoms than controls (*k* = 2, *n* = 89, SMD = 0.677, 95% CI = 0.249–1.105, *p* = .002) (Figure 3). Again, when included in the meta‐analysis, omega‐3 PUFA was the only intervention that reduced total symptoms significantly more than control (SMD = 0.64, *p* = .005). The heterogeneity across the studies was not significant (*Q* = 0.422, *I* ^2^ = 0.000, *p* = .517) and was unlikely to be important (Higgins et al., 2024). As the evidence for this outcome only came from RCTs, the certainty for evidence started as high quality however was downgraded to moderate due to the results being analysed using a random effects model.

### Depressive symptoms

Two studies were included in the meta‐analysis for the outcome of depressive symptoms (Amminger et al., 2010; Woods et al., 2013). Preventative interventions (including omega‐3 PUFA and glycine) were not significantly more effective at reducing depression symptoms than controls (*k* = 2, *n* = 89, SMD = 0.936, 95% CI = 0.788–2.661, *p* = .287) (Figure S2). When included in the meta‐analysis, the only individual intervention to reduce depressive symptoms significantly more than control was glycine (SMD = 2.04, *p* = .02). The heterogeneity across the studies was significant (*Q* = 4.022, *I* ^2^ = 75.137, *p* = .045) and indicated considerable heterogeneity (Higgins et al., 2024). Again, as the evidence for this outcome only came from RCTs, the certainty for evidence started as high quality however was downgraded to moderate due to the results being analysed using a random effects model as heterogeneity was high.

### Functioning

Four studies were included in the meta‐analysis for the outcome of functioning (Amminger et al., 2010; Grano et al., 2016; McFarlane et al., 2015; Stain et al., 2016). Preventative interventions (including omega‐3 PUFA, FACT, cognitive behavioural therapy and family and community oriented integrative treatment [FCTM]) were significantly more effective at improving global functioning than controls (*k* = 4, *n* = 486, SMD = 0.944, 95% CI = 0.052–1.836, *p* = .038) (Figure 3). When included in the meta‐analysis, omega‐3 PUFA (SMD = 0.72, *p* = .002), FCTM (SMD = 0.67, *p* = .015) and FACT (SMD = 2.04, *p* = <.001) all improved functioning significantly more than controls. The heterogeneity across the studies was significant (*Q* = 49.598, *I* ^2^ = 93.951, *p* < .001) and indicated considerable heterogeneity (Higgins et al., 2024). Asymmetry in the funnel plot for the analysis and a significant Egger's test indicated publication bias (*t* = 5.57, *p* = .031) (Figure S6 and Table S8). To correct for publication bias, we used a trim and fill method, and the adjusted results were: SMD = 1.16, 95% CI = 0.402–1.918. As the evidence for this outcome came from RCTs and one observational study, the certainty of evidence started as moderate due to indirectness and high heterogeneity. It was further downgraded to low due to publication bias.

Publication bias was not detected for any of the other outcomes in the funnel plots (Figures S3–S5) nor Egger's test (Table S8). However, Egger's tests and funnel plots could not be produced for the outcomes of total symptoms and depressive symptoms because these analyses involved too few studies.

### Narrative synthesis of studies unable to be included in meta‐analysis

The following studies could not be included in the meta‐analysis because they either used the same sample as a study already included in the meta‐analyses (Amminger et al., 2013, 2015; Mossaheb et al., 2013; Woods et al., 2013), because the intervention of interest was compared against an active control group (another intervention) (Addington et al., 2023; Bowie et al., 2012; Cornblatt et al., 2007; Holzer et al., 2014; Janssen et al., 2021; Miklowitz et al., 2014; O'Brien et al., 2014; O'Brien Cannon et al., 2022; Urben et al., 2012; Waite et al., 2023), or the study had a naturalistic design and so did not compare the intervention of interest against a control (Brazzale et al., 2018; McAusland & Addington, 2018; O'Brien et al., 2007; Pitzianti et al., 2019; Woodberry et al., 2021; Woods et al., 2007).

For the outcome of transition to psychosis, five other studies that were not included in the meta‐analysis evaluated whether participants had transitioned to a first episode of psychosis by follow up after the intervention (Addington et al., 2023; Amminger et al., 2013, 2015; Cornblatt et al., 2007; Miklowitz et al., 2014). In these studies, Omega‐3 PUFA still outperformed the placebo at 6.7‐year follow‐up after the intervention (Amminger et al., 2015), anti‐depressants reduced risk of transition to psychosis significantly more than antipsychotics (Cornblatt et al., 2007) and family focused therapy (FFT) outperformed a psychoeducational control at reducing transition to psychosis risk (Miklowitz et al., 2014). Cognitive behavioural social skills training did not outperform a control of supportive therapy (Addington et al., 2023). As the evidence for this outcome came from RCTs and one observational study, the certainty of evidence started as moderate and was further downgraded to low due to two studies having a high risk of bias.

For the outcome of attenuated positive symptoms, 14 other studies that were not included in the meta‐analysis evaluated whether participants' positive psychosis symptoms had reduced (Addington et al., 2023; Amminger et al., 2013, 2015; Bowie et al., 2012; Brazzale et al., 2018; Cornblatt et al., 2007; Grano et al., 2016; Janssen et al., 2021; McAusland & Addington, 2018; Miklowitz et al., 2014; Mossaheb et al., 2013; O'Brien et al., 2007; Stain et al., 2016; Woodberry et al., 2021; Woods et al., 2007). Whilst most of the studies reported change in positive symptoms over time, the only interventions that showed a significant effect over the control groups were FFT compared to a psychoeducational control (Miklowitz et al., 2014) and omega‐3 PUFA compared to sucrose placebo (Amminger et al., 2013, 2015). As the majority of evidence for this outcome came from observational studies, the certainty of evidence started as low and was further downgraded to very low due to multiple studies having a high risk of bias.

For the outcome of negative symptoms, eight other studies that were not included in the meta‐analysis evaluated whether participants' negative symptoms had reduced (Addington et al., 2023; Amminger et al., 2013, 2015; Grano et al., 2016; Miklowitz et al., 2014; O'Brien et al., 2007; Stain et al., 2016; Woods et al., 2007). Whilst aripiprazole antipsychotic (Woods et al., 2007) or group, community or family‐based interventions (Addington et al., 2023; Miklowitz et al., 2014; O'Brien et al., 2007;Grano et al., 2016) produced a significant change in negative symptoms over time, Omega‐3 PUFA was the only intervention that outperformed sucrose placebo (Amminger et al., 2013) and this persisted until 6.7 years after the intervention (Amminger et al., 2015). As evidence came from a mixture of RCTs and observational studies, the certainty of evidence started as moderate and was further downgraded to low due to multiple studies having a high or moderate risk of bias.

For the outcome of total symptoms five other studies that were not included in the meta‐analysis evaluated whether participants' total symptoms had reduced (Amminger et al., 2013, 2015; Holzer et al., 2014; Waite et al., 2023; Woods et al., 2007, 2013). Whilst SleepWell (Waite et al., 2023), aripiprazole antipsychotic (Woods et al., 2007), glycine (Woods et al., 2013) and cognitive remediation interventions (Holzer et al., 2014) reported reduction over time in total symptoms, Omega‐3 PUFA was the only intervention that outperformed the sucrose placebo control (Amminger et al., 2013) and this persisted until 6.7 years after the intervention (Amminger et al., 2015). As evidence came from a mixture of RCTs and observational studies, the certainty of evidence started as moderate and was further downgraded to low due to multiple studies having a high or moderate risk of bias.

For the outcome of depressive symptoms nine other studies that were not included in the meta‐analysis evaluated whether participants' depressive symptoms had reduced (Addington et al., 2023; Amminger et al., 2013, 2015; Bowie et al., 2012; Grano et al., 2016; McAusland & Addington, 2018; Stain et al., 2016; Waite et al., 2023; Woods et al., 2007). Many of the interventions produced a reduction in depressive symptoms over time including CBT based interventionsnon‐directive reflective listening (NDRL; Stain et al., 2016), biofeedback (McAusland & Addington, 2018), antidepressants (Bowie et al., 2012) and antipsychotics (Bowie et al., 2012; Woods et al., 2007). Omega‐3 (Amminger et al., 2013), SleepWell (Waite et al., 2023), family and community integrative treatment (FCTM; Grano et al., 2016) were the only interventions that outperformed the controls of sucrose placebo, usual care and treatment as usual, respectively. As the majority of evidence for this outcome came from observational studies, the certainty of evidence started as low and was further downgraded to very low due to multiple studies having a high or moderate risk of bias.

For the outcome of functioning, 11 other studies that were not included in the meta‐analysis evaluated whether participants' functioning had improved (Addington et al., 2023; Amminger et al., 2013, 2015; Holzer et al., 2014; Janssen et al., 2021; McAusland & Addington, 2018; Miklowitz et al., 2014; O'Brien et al., 2007; Woodberry et al., 2021; Woods et al., 2007, 2013). Whilst cognitive behavioural social skills (Addington et al., 2023). aripiprazole (Woods et al., 2007), omega‐3 PUFA (Amminger et al., 2013) and psychoeducational multifamily groups (PMFG; O'Brien et al., 2007) increased functioning over time, only omega‐3 PUFA outperformed the control of sucrose placebo. As the majority of evidence for this outcome came from observational studies, the certainty of evidence started as low and was further downgraded to very low due to multiple studies having a high or moderate risk of bias.

The narrative synthesis and discussion of findings on the secondary outcomes are reported in the supporting information (Results S1).

## Discussion

To our knowledge, this is the first meta‐analysis to assess the effectiveness of interventions for CHR‐P focussing on children and adolescents across multiple outcomes impacting prognosis. The results of the meta‐analysis indicated that preventative interventions were more effective than no intervention or placebo at reducing positive, negative and total symptoms and improving functioning. However, they were not effective at significantly reducing transition to psychosis risk or depression symptoms. The largest effect size was seen for improvement in functioning (SMD = 0.944, 95% CI 0.052–1.836) and the smallest effect size was for attenuated positive symptoms (SMD = 0.379, 95% CI 0.055–0.703).

### Transition to psychosis risk

Previous meta‐analyses including adults found that, in general, preventative interventions were effective at reducing transition to psychosis risk (Mei et al., 2021; Schmidt et al., 2015; Van Der Gaag et al., 2013). However, the findings of the current study suggest that preventative interventions may not be as effective for CHR‐P children and adolescents in mitigating this risk. This finding should be interpreted with caution as the modest rates of transition to psychosis in the included studies may have had an impact on the ability to detect small to moderate effect sizes for the interventions. This variation in the effectiveness of preventative intervention based on age aligns with findings from an earlier meta‐analysis which suggested that among adult participants, these preventative interventions were less effective for younger individuals compared to older ones at reducing risk of transition to psychosis (Schmidt et al., 2015). CHR‐P children and adolescents have a poorer prognosis than CHR‐P adults such that the earlier the onset of prodromal symptoms, the longer the period of risk of transition to psychosis (Dominguez et al., 2013). This extended period increases the chronicity of social stress and disruption to functioning which further elevates their risk of transitioning to psychosis (Catalan et al., 2021). Despite this, network meta‐analyses in adult populations have not identified one intervention to be superior to others (Davies, Cipriani, et al., 2018) so further research is required to identify truly effective interventions.

### Attenuated positive psychotic symptoms

Across meta‐analyses including adults, preventative interventions (when psychological and pharmacological interventions were pooled) were more effective than any controls at reducing positive symptoms with a small effect size after 12 months but not at any other time point (Mei et al., 2021). In a similar way in the current meta‐analysis of studies only including children and adolescent participants, when pharmacological (omega‐3 and glycine) and psychological (FACT) preventative interventions were pooled there was a significant effect on reducing positive symptoms over placebo or no intervention and this effect was larger for children and adolescents (SMD = 0.38) than for adults. However when pooled separately, pharmacological and psychological preventative interventions were not effective at reducing positive symptoms (Mei et al., 2021; Stafford et al., 2013). The larger effect size seen in the current meta‐analysis may have been because previous meta‐analyses including adults compared any experimental intervention with any control group including active controls of other interventions (Mei et al., 2021; Stafford et al., 2013) whereas in the current meta‐analysis, interventions were compared against placebo or no intervention which was expected to produce a greater effect. This was supported by network meta‐analyses, which found there was no evidence to favour any one intervention over other active interventions to reduce positive symptoms (Davies, Radua, et al., 2018; Devoe, Farris, Townes, & Addington, 2019a).

### Negative psychotic symptoms

Few meta‐analyses including adults assessed the efficacy of preventative interventions at reducing CHR‐P participants' negative symptoms (Devoe et al., 2018; Mei et al., 2021). When psychological and pharmacological interventions were pooled, there was no significant effect of interventions over any controls on reducing negative symptoms across any time point (Mei et al., 2021). This contradicts the findings of the current meta‐analysis which found a significant effect of preventative interventions (when pharmacological and psychological interventions were pooled) compared to placebo or no intervention (SMD = 0.58, 95% CI 0.187–0.980). These results are clinically relevant due to the high proportion (around 80%) of negative symptoms of CHR‐P children and adolescents and their association with poor prognosis (Salazar De Pablo et al., 2023). However, the disparity in the efficacy of interventions in improving negative symptoms between children and adults may reflect a lower true‐positive rate for psychosis in CHR‐P children compared to adults. Additionally, there may be an overlap between negative psychotic symptoms and symptoms of other developing mental health disorders in children which may be more responsive to intervention (Gerstenberg et al., 2016).

### Total symptoms

To our knowledge, no previous meta‐analysis including adults has evaluated the efficacy of preventative interventions in reducing the total number of prodromal psychosis symptoms.

### Depressive symptoms

A previous meta‐analyses including adults found that when psychological and pharmacological interventions were pooled and compared to any control group, preventative interventions for CHR‐P participants were not significantly more effective at reducing depressive symptoms than controls at any time point (Mei et al., 2021). The combined effects of pharmacological interventions (including olanzapine, risperidone, and amisulpride) were also not significantly more effective at reducing depression symptoms than controls of no active intervention or placebo (Stafford et al., 2013). A similar result was found in the current meta‐analysis in children and adolescents such that the combined effects of pharmacological preventative interventions were not significantly different to placebo at reducing depression.

### Functioning

A previous meta‐analysis including adults found that the combined effects of pharmacological and psychological interventions (including CBT, olanzapine, family‐focused therapy, and amisulpride) were significantly more effective than controls at improving functioning at short term (up to 6 months) follow‐up but only after excluding a study of low quality. At all other time points, the combined effects of these preventative interventions were not significantly more effective than controls (Schmidt et al., 2015). Similarly, in another meta‐analysis including adult samples, when psychological and pharmacological interventions were pooled, preventative interventions were not significantly more effective at improving functioning than controls at any time point. The inefficacy of preventative interventions at improving functioning compared to controls remained when psychological and pharmacological interventions were pooled separately across any time point (Mei et al., 2021). Notably, this contradicted findings in the current meta‐analysis such that preventative interventions significantly improved functioning compared to controls with a large effect size. Again, this disparity may have been because these previous meta‐analyses compared any experimental intervention with any control group including active controls (other interventions) whereas in the current meta‐analysis, interventions were compared against placebo or no intervention. Alternatively, preventative interventions may be uniquely effective at improving functioning for CHR‐P children and adolescents since children and adolescents are still developing, they may have more to gain in terms of functionality through interventions than adults.

Egger's test revealed there was publication bias in the meta‐analysis for functioning. We used the trim and fill method to correct for potentially lacking articles and we found that the effect size in favour of the intervention increased suggesting that potentially missing studies also favoured the intervention over control so publication bias was unlikely to impact the results (Shi & Lin, 2019).

### Preliminary evidence of individual interventions within the meta‐analysis

As for individual interventions within the meta‐analysis, there is preliminary evidence to suggest that omega‐3 PUFA was effective at improving risk of transition to psychosis, positive symptoms, negative symptoms, total symptoms and functioning however not effective at improving depression (Amminger et al., 2010). The effect sizes for omega‐3 PUFA were all medium and the effect on functioning was the largest. Whilst in meta‐analyses including adults, omega‐3 PUFA did not significantly outperform controls at improving transition to psychosis risk, attenuated positive symptoms, negative symptoms or functioning (Cadenhead et al., 2017; Devoe et al., 2018, 2019a; Devoe, Farris, Townes, & Addington, 2019b; McGorry et al., 2017), the results of the current study reinforce suggestions that omega‐3 is more effective when given as early as possible in the course of psychotic disorders (Amminger et al., 2010). Notably, only one study assessed the efficacy of omega‐3 PUFA in children and adolescents so more replications of this result are needed.

Regarding psychological preventative interventions, FCTM (Grano et al., 2016) and FACT (McFarlane et al., 2015) significantly improved functioning with a medium and very large effect size, respectively, however this result was not seen in meta‐analyses on CHR‐P adults assessing the efficacy of family therapy and integrated psychotherapy. The inefficacy of CBT at improving functioning (Stain et al., 2016) was also seen in adult samples (Hutton & Taylor, 2014).

### Limitations

There were several limitations of the current meta‐analysis and systematic review. Principally, a limited number of studies (and hence, preventative interventions) were included in the current meta‐analysis, due to limited available evidence. Another limitation is that meta‐analytical regressions could not be conducted based on length of treatment, follow‐up period or study quality as each outcome had too few included studies to conduct these subanalyses. These subanalyses may have provided further information about the mechanisms of efficacy for these preventative interventions and this is a potential avenue for future research should more intervention studies in children be conducted. Furthermore, there was great heterogeneity in the quality of the included studies and due to the very limited number of studies included for each outcome, sensitivity analyses excluding studies of low quality could not be conducted. A limitation that comes from the limited number of studies included for each outcome is that we could only compare interventions against an inactive control group and we could not compare the efficacy of different pharmacological and psychological interventions against each other. Furthermore, only a few trials assessed outcomes at long‐term follow‐up (>1 year) and this may impact findings for transition to psychosis which requires time to capture. Finally, most symptoms were evaluated by psychometric instruments, which were heterogeneous too.

### Clinical implications

There are also several clinical implications and suggestions for future research that arose from this review. First, although preventative interventions for CHR‐P children and adolescents were not able to reduce the risk of transition to psychosis, they did reduce prodromal symptoms and improve functioning which also predict prognosis and impacts quality of life. This supports the need for early intervention in psychosis services and clinicians to be more comprehensive in CHR‐P treatment plans. Furthermore, where pharmacological and psychological interventions had similar effects on outcomes, clinicians should first explore the psychological interventions to avoid adverse effects of pharmacological treatments for children and adolescents who are subsyndromal. However, acceptability, adherence and adverse effects are all additional factors that determine intervention effectiveness which were not encompassed within this review and future studies may wish to explore this given that adverse effects would highly influence treatment decisions in children and adolescents who are subsyndromal.

## Conclusions

In conclusion, this meta‐analysis provides evidence that preventative interventions (when pharmacological and psychological interventions were pooled) were effective at reducing positive, negative and total symptoms and improving functioning compared to no intervention but were ineffective at reducing transition to psychosis risk and depression symptoms. There are disparities compared to adult meta‐analyses such that in adults preventative interventions were effective at reducing transition to psychosis risk but were not effective at reducing negative symptoms and improving functioning. There is preliminary evidence that omega‐3 PUFA may be uniquely effective as a preventative intervention for CHR‐P children and adolescents but not for adults as existing literature has shown, however, this evidence was from only one study. Integrated family and community interventions were also effective at improving functioning in CHR‐P children and adolescents but not in adults. Further studies on preventative interventions for CHR‐P children and adolescents are needed before definitive conclusions can be drawn.

## Conflict of interest statement

Dr Salazar de Pablo has received honoraria from Lundbeck Foundation, Janssen Cilag and Menarini. Dr Salazar de Pablo is also a member of the Editorial Board for The Child and Adolescent Mental Health Journal. Dr Aymerich has received personal fees or grants from Janssen Cilag and Neuraxpharm outside the current work.

## Author contributions

Dr Javier de Otazu Olivares acted as the second reviewer during the screening of studies identified from the database searches and during the process of data extraction and was involved in the final approval of the version to be published. Dr Ana Catalan and Dr Claudia Aymerich were involved in reviewing, editing and the critical revision of the study. They also were involved in the final approval of the version to be published. Dr Gonzalo Salazar de Pablo acted as a third independent reviewer during the screening and data extraction processes and was involved in idea conception, drafting of the study, quantitative data analysis and the final approval of the version to be published.

## Funding information

No separate funding required for this article.

## Ethical information

No ethical approval was sought for this paper as this was a systematic review and meta‐analysis of data that was publicly accessible.

## Supporting information


**Methods S1.** Validated instruments to assess CHR‐P.
**Methods S2.** Definitions and description of mental health outcomes included in the current systematic review and meta‐analysis.
**Figure S1.** ROB‐2 quality assessment results.
**Figure S2.** Forest plot for depression symptoms outcome.
**Figure S3.** Funnel plot for transition to psychosis outcome.
**Figure S4.** Funnel plot for positive symptoms outcome.
**Figure S5.** Funnel plot for negative symptoms outcome.
**Figure S6.** Funnel plot for global functioning outcome.
**Table S1.** PRISMA guidelines.
**Table S2.** PRISMA Abstract guidelines.
**Table S3.** MOOSE checklist.
**Table S4.** Data extraction table with additional information not included in study characteristics table.
**Table S5.** ROBINS‐I quality assessment results.
**Table S6.** ROB2 assessment for all included studies for each outcome.
**Table S7.** Results of heterogeneity analysis and random effects model.
**Table S8.** Publication bias: Egger's test results.

## Data Availability

Data sharing is not applicable to this article as no new data were created or analysed in this study.
