# Supplementary material for: Review: Efficacy of preventative interventions for children and adolescents at clinical high risk of psychosis – a systematic review and meta‐analysis of intervention studies
Source: Child Adolesc Ment Health. 2024 Dec 17;30(1):66–82. doi: 10.1111/camh.12755 (PMC11754713; doi:10.1111/camh.12755)
Supplement: Supplementary file 1 — Methods S1. Validated instruments to assess CHR‐P. Methods S2. Definitions and description of mental health outcomes included in the current systematic review and meta‐analysis. Figure S1. ROB‐2 quality assessment results. Figure S2. Forest plot for depression symptoms outcome. Figure S3. Funnel plot for transition to psychosis outcome. Figure S4. Funnel plot for positive symptoms outcome. Figure S5. Funnel plot for negative symptoms outcome. Figure S6. Funnel plot for global functioning outcome. Table S1. PRISMA guidelines. Table S2. PRISMA Abstract guidelines. Table S3. MOOSE checklist. Table S4. Data extraction table with additional information not included in study characteristics table. Table S5. ROBINS‐I quality assessment results. Table S6. ROB2 assessment for all included studies for each outcome. Table S7. Results of heterogeneity analysis and random effects model. Table S8. Publication bias: Egger's test results. [file CAMH-30-66-s001.docx]

**Supporting Information**

Contents:

Table S1: PRISMA guidelines………………………………………………………………Page 2

Table S2: PRISMA Abstract guidelines…………………………………………………….Page 5

Table S3: MOOSE checklist…………………………………………………………………Page 6

Methods S1: Instruments used to assess CHR-P…………………………………………….Page 8

Methods S2: Definitions and description of mental health outcomes………………………Page 8

Table S4: Detailed data extraction table…………………………………………………….Page 9

Figure S1: ROB-2 quality assessment results……………………………………………….Page 16

Table S5: ROBINS-I quality assessment results…………………………………………….Page 17

Table S6: ROB-2 assessment for all included studies for each outcome……………………Page 18

Table S7: Results of heterogeneity analyses………………………………………………..Page 22

Figure S2: Forest plot for depression symptoms outcome.………………………………….Page 23

Table S8: Publication bias: Egger’s test results……………………………………………...Page 24

Figure S3: Funnel plot for transition to psychosis outcome…………………………………Page 25

Figure S4: Funnel plot for positive symptoms outcome……………………………………..Page 26

Figure S5: Funnel plot for negative symptoms outcome…………………………………….Page 27

Figure S6: Funnel plot for global functioning outcome………………………………………Page 28

Results S1: Narrative synthesis and discussion of secondary outcomes……………………...Page 29

**This supplementary material has been provided by the authors to give readers additional information about their work.**

**Table S1.** PRISMA guidelines.

| **Section and Topic** | **Item #** | **Checklist item** | **Location where item is reported** |
| --- | --- | --- | --- |
| **TITLE** | | |  |
| Title | 1 | Identify the report as a systematic review. | Page 1 |
| **ABSTRACT** | | |  |
| Abstract | 2 | See the PRISMA 2020 for Abstracts checklist. | Page 2 |
| **INTRODUCTION** | | |  |
| Rationale | 3 | Describe the rationale for the review in the context of existing knowledge. | Page 4 |
| Objectives | 4 | Provide an explicit statement of the objective(s) or question(s) the review addresses. | Page 3 and 4 |
| **METHODS** | | |  |
| Eligibility criteria | 5 | Specify the inclusion and exclusion criteria for the review and how studies were grouped for the syntheses. | Page 6, 8 and 9 |
| Information sources | 6 | Specify all databases, registers, websites, organisations, reference lists and other sources searched or consulted to identify studies. Specify the date when each source was last searched or consulted. | Page 6 |
| Search strategy | 7 | Present the full search strategies for all databases, registers and websites, including any filters and limits used. | Page 6 |
| Selection process | 8 | Specify the methods used to decide whether a study met the inclusion criteria of the review, including how many reviewers screened each record and each report retrieved, whether they worked independently, and if applicable, details of automation tools used in the process. | Page 6 and 7 |
| Data collection process | 9 | Specify the methods used to collect data from reports, including how many reviewers collected data from each report, whether they worked independently, any processes for obtaining or confirming data from study investigators, and if applicable, details of automation tools used in the process. | Page 7 |
| Data items | 10a | List and define all outcomes for which data were sought. Specify whether all results that were compatible with each outcome domain in each study were sought (e.g. for all measures, time points, analyses), and if not, the methods used to decide which results to collect. | Page 7 |
|  | 10b | List and define all other variables for which data were sought (e.g. participant and intervention characteristics, funding sources). Describe any assumptions made about any missing or unclear information. | Page 7 |
| Study risk of bias assessment | 11 | Specify the methods used to assess risk of bias in the included studies, including details of the tool(s) used, how many reviewers assessed each study and whether they worked independently, and if applicable, details of automation tools used in the process. | Page 7, 8 |
| Effect measures | 12 | Specify for each outcome the effect measure(s) (e.g. risk ratio, mean difference) used in the synthesis or presentation of results. | Page 8 |
| Synthesis methods | 13a | Describe the processes used to decide which studies were eligible for each synthesis (e.g. tabulating the study intervention characteristics and comparing against the planned groups for each synthesis (item #5)). | Page 7 |
|  | 13b | Describe any methods required to prepare the data for presentation or synthesis, such as handling of missing summary statistics, or data conversions. | Page 8 |
|  | 13c | Describe any methods used to tabulate or visually display results of individual studies and syntheses. | Page 8 |
|  | 13d | Describe any methods used to synthesize results and provide a rationale for the choice(s). If meta-analysis was performed, describe the model(s), method(s) to identify the presence and extent of statistical heterogeneity, and software package(s) used. | Page 8 |
|  | 13e | Describe any methods used to explore possible causes of heterogeneity among study results (e.g. subgroup analysis, meta-regression). | Page 8 |
|  | 13f | Describe any sensitivity analyses conducted to assess robustness of the synthesized results. | N/A |
| Reporting bias assessment | 14 | Describe any methods used to assess risk of bias due to missing results in a synthesis (arising from reporting biases). | Page 7, 8 |
| Certainty assessment | 15 | Describe any methods used to assess certainty (or confidence) in the body of evidence for an outcome. | Page 7.8 |
| **RESULTS** | | |  |
| Study selection | 16a | Describe the results of the search and selection process, from the number of records identified in the search to the number of studies included in the review, ideally using a flow diagram. | Page 9 |
|  | 16b | Cite studies that might appear to meet the inclusion criteria, but which were excluded, and explain why they were excluded. | Page 9 |
| Study characteristics | 17 | Cite each included study and present its characteristics. | Page 10 |
| Risk of bias in studies | 18 | Present assessments of risk of bias for each included study. | Page 10, eFigure 1 and eTable 5, 6 |
| Results of individual studies | 19 | For all outcomes, present, for each study: (a) summary statistics for each group (where appropriate) and (b) an effect estimate and its precision (e.g. confidence/credible interval), ideally using structured tables or plots. | Pages 10-15 |
| Results of syntheses | 20a | For each synthesis, briefly summarise the characteristics and risk of bias among contributing studies. | Pages 10-15 |
|  | 20b | Present results of all statistical syntheses conducted. If meta-analysis was done, present for each the summary estimate and its precision (e.g. confidence/credible interval) and measures of statistical heterogeneity. If comparing groups, describe the direction of the effect. | Page 10-15 and eTable 7 |
|  | 20c | Present results of all investigations of possible causes of heterogeneity among study results. | Pages 10-15 |
|  | 20d | Present results of all sensitivity analyses conducted to assess the robustness of the synthesized results. | N/A |
| Reporting biases | 21 | Present assessments of risk of bias due to missing results (arising from reporting biases) for each synthesis assessed. | N/A |
| Certainty of evidence | 22 | Present assessments of certainty (or confidence) in the body of evidence for each outcome assessed. | Pages 10-15 |
| **DISCUSSION** | | |  |
| Discussion | 23a | Provide a general interpretation of the results in the context of other evidence. | Pages 15-19 |
|  | 23b | Discuss any limitations of the evidence included in the review. | Page 20 and 21 |
|  | 23c | Discuss any limitations of the review processes used. | Page 20 and 21 |
|  | 23d | Discuss implications of the results for practice, policy, and future research. | Page 21 |
| **OTHER INFORMATION** | | |  |
| Registration and protocol | 24a | Provide registration information for the review, including register name and registration number, or state that the review was not registered. | Page 6 |
|  | 24b | Indicate where the review protocol can be accessed, or state that a protocol was not prepared. | Page 6 |
|  | 24c | Describe and explain any amendments to information provided at registration or in the protocol. | N/A |
| Support | 25 | Describe sources of financial or non-financial support for the review, and the role of the funders or sponsors in the review. | Page 23 |
| Competing interests | 26 | Declare any competing interests of review authors. | Page 23 |
| Availability of data, code and other materials | 27 | Report which of the following are publicly available and where they can be found: template data collection forms; data extracted from included studies; data used for all analyses; analytic code; any other materials used in the review. | eTable 4 |

**Table S2.** PRISMA Abstract guidelines.

*
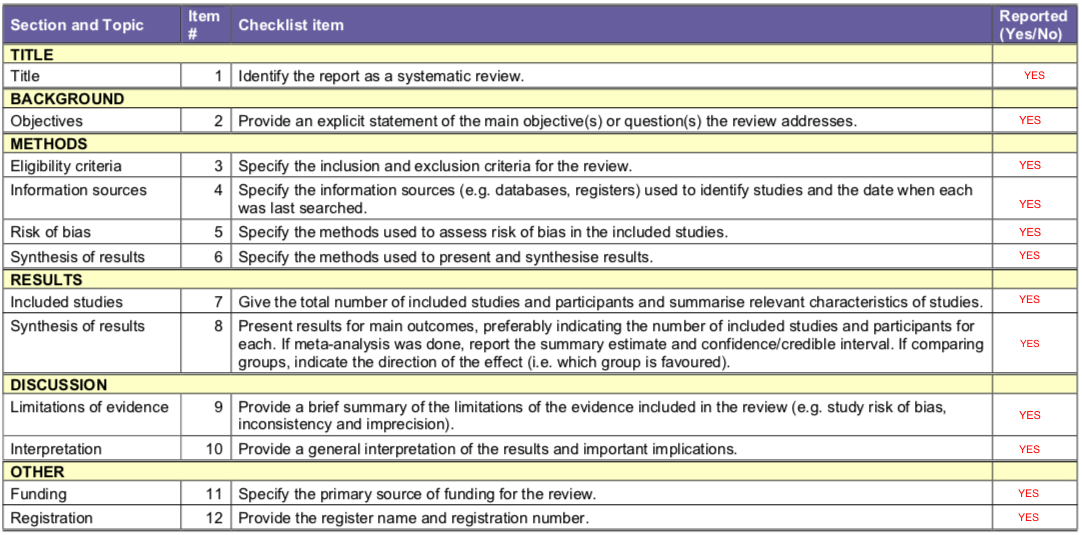
*

**Table S3.** MOOSE checklist.

| *Criteria* | | *Description of how criteria were met in the meta-analysis* |
| --- | --- | --- |
| Reporting of background | |  |
| ✓ | Problem definition | No meta-analysis has evaluated the efficacy of preventative interventions for CHR-P children and adolescents across multiple mental health outcomes. |
| ✓ | Hypothesis statement | We hypothesised that preventative interventions would be effective but have smaller effect size than preventative interventions for CHR-P adults. |
| ✓ | Description of study outcomes | Studies were described in study characteristics table |
| ✓ | Type of exposure or intervention used | Any preventative intervention group |
| ✓ | Type of study design | Studies of intervention |
| ✓ | Study population | Children and adolescents at CHR-P. |
| Reporting of search criteria | |  |
| ✓ | Qualifications of searchers | Credentials of authors were included in authors list |
| ✓ | Search strategy including time period included in the synthesis and key words | Search strategy is detailed in the methods |
| ✓ | Databases and registries searched | Ovid MEDLINE, APA PsycInfo and Web of Science |
| ✓ | Use of hand searching | Reviews were hand searched using backwards citation chasing. |
| ✓ | List of citations located and excluded including justifications | Results of search and screening are detailed in the PRISMA flowchart |
| ✓ | Methods of addressing articles published in languages other than English | Only articles in English were slected. |
| ✓ | Method of handling abstracts and unpublished studies | Cochrane CENTRAL and ProQuest dissertations and theses to search for grey literature |
| ✓ | Description of any contact with authors | No contact with authors was required |
| Reporting of methods should include | |  |
| ✓ | Description of relevance or appropriateness of studies assembled for assessing the hypothesis to be tested | Detailed inclusion and exclusion criteria were included in the methods section. |
| ✓ | Rationale for the selection and coding of data | Data extracted was relevant to the study outcomes. |
| ✓ | Assessment of confounding | We did not investigate modifiers of treatment response. |
| ✓ | Assessment of study quality, including blinding of quality assessors, stratification or regression on possible predictors of study results | Assessment of study quality is included in the results section. |
| ✓ | Assessment of heterogeneity | Heterogeneity was assessed using the I^2^ index |
| ✓ | Description of statistical methods in sufficient detail to be replicated | A random effects model was used. Heterogeneity among study point estimates was assessed using Q statistics. The proportion of total variability in the effect size estimates was evaluated with the I^2^ index |
| ✓ | Provision of appropriate tables and graphics | PRISMA flowchart, study characteristics table, forest plots, funnel plots to assess publication bias, tables of heterogeneity results, tables of Egger’s test results and quality assessment results were included. |
| Reporting of results should include | |  |
| ✓ | Table summarising individual study estimates and overall estimate | Reported in the results and supplementary materials |
| ✓ | Table giving descriptive information for each study | Reported in the study characteristics table. |
| ✓ | Results of sensitivity testing | Subgroup analysis could not be conducted because of the small number of studies reporting each outcome. |
|  | Indication of statistical uncertainty of findings | Reported in the results. |
| Reporting of discussion should include | |  |
| ✓ | Quantitative assessment of bias | Assessment of publication bias was assessed using Egger’s test and funnel plots which are seen in supplementary materials. |
| ✓ | Justification for exclusion | Justification for exclusion was described in discussion as well as methods. |
| ✓ | Assessment of quality of included studies | Included in the study characteristics table and supplementary materials. |
| Reporting of conclusions should include | |  |
| ✓ | Consideration of alternative explanations for observed results | Assessed and proposed in the discussion. |
| ✓ | Generalisation of the conclusions | Addressed in the discussion section. |
| ✓ | Guidelines for future research | Addressed in the discussion section. |
| ✓ | Disclosure of funding source | Funding source was specified. |

**Methods S1.** Validated instruments to assess CHR-P.

DSM-5 Attenuated Psychosis Syndrome Clinical criteria (APS) (Salazar de Pablo et al., 2020)

Comprehensive Assessment of At-Risk Mental States (CAARMS) (Yung et al., 2005),

Structured Interview for Psychosis-risk Syndromes (SIPS) (Miller et al., 1999),

Bonn Scale for the Assessment of Basic Symptoms (BSABS) (Vollmer-Larsen et al., 2007)),

Basel Screening Instrument for Psychosis (BSIP) (Riecher-Rössler et al., 2008),

Schizophrenia Proneness Instrument (SPI) (Fux et al., 2013)  - Adult (SPI-A) and Child and Youth (SPI-CY) version -

Positive and Negative Syndrome Scale (PANSS) (Kay et al., 1987),

Scale for the Assessment of Negative Symptoms (SANS) (Andreasen, 1989),

Brief Psychiatric Rating Scale (BPRS) (Overall and Gorham, 1988)

Early Recognition Inventory (ERIraos), (Rausch et al., 2013)

**Methods S2*.*** Definitions and description of mental health outcomes included in the current systematic review and meta-analysis.

In the majority of included studies these baseline and follow up assessments were conducted by the researchers or Masters or PHD level clinicians.

**Rate of transition to psychosis by follow up**: Assessed using Positive and Negative Syndrome Scale (PANSS), Structured interview for psychosis-risk syndromes (SIPS/SOPS), Comprehensive Assessment of At Risk Mental States (CAARMS), Kiddie Schedule for Affective Disorders and Schizophrenia (K-SADS).

**Change in positive prodromal psychotic symptoms:** Assessed using Positive and Negative Syndrome Scale (PANSS), Structured interview for psychosis-risk syndromes (SIPS/SOPS). In the majority of included studies these baseline and follow up assessments were conducted by the researchers.

**Change in negative prodromal psychotic symptoms**: Assessed using Positive and Negative Syndrome Scale (PANSS), Structured interview for psychosis-risk syndromes (SIPS/SOPS)

**Change in total number of prodromal psychotic symptoms**: Assessed using Positive and Negative Syndrome Scale (PANSS), Structured interview for psychosis-risk syndromes (SIPS/SOPS)

**Change in depressive symptoms**: Assessed using Montgomery-Asberg Depression Rating Scale (MADRS), Beck Depression Inventory (BDI), Children’s Depression Rating Scale (CDRS).

**Change in functioning**: Assessed using the Global Assessment of Functioning (GAF)

**Table S4.** Data extraction table with additional information not included in study characteristics table.

| **First author and year** | **Country** | **Intervention versus control** | **Components of psychological intervention** | **Number of participants and mean age (SD)** | **Percentage males** | **Scales used to assess outcomes** | **Length of intervention versus follow up** |
| --- | --- | --- | --- | --- | --- | --- | --- |
| Addington, 2023 | USA and Canada | Cognitive behavioural social skills training (CBSST) versus control group of group supportive therapy (ST). | Targeting functional outcomes, training new social skills and target thinking patterns eg: low self-efficacy, defeatist beliefs | CBSST: n=70, age=17.36 (4.01)  ST: n=82, age= 17.49 (4.12) | 45.40% | Attenuated positive symptoms (SOPS), negative symptoms (SOPS), depression (Calgary Depression scale for schizophrenia), functioning: social and role (Global functioning scales GF:S and GF:R). | 18 weeks, end of treatment 18 weeks and then 12 month follow up. |
| Amminger, 2010 | Austria | Omega-3 PUFA vs coconut oil placebo | n/a | Omega-3: n=41, age=16.8 (2.4)  Placebo: n=40, age=16.0 (1.7) | 33% | Conversion to psychosis *(PANSS severity thresholds),* Positive, negative and total psychotic symptoms *(PANSS),* Depressive symptoms *(MADRS),* Functioning *(GAF)* | 3 months; 3, 6, 12 months |
| Amminger, 2013 | Australia | Omega-3 PUFA for CHR-P with BPD vs coconut oil placebo | n/a | Omega-3: n=8, age for whole group= 16.2 (2.1)  Placebo: n=7 | 6.70% | Positive and negative psychotic symptoms as well as BPD symptom subscale (PANSS), Depression symptoms *(MADRS),* Functioning *(GAF)* | 12 weeks; 12 weeks |
| Amminger, 2015 | Austria | Omega-3 PUFA vs coconut oil placebo | n/a | Omega-3: n=41, age=16.8 (2.4)  Placebo: n=40, age=16.0 (1.7) | 33% | Conversion to psychosis *(PANSS severity thresholds),* Positive, negative and total psychotic symptoms *(PANSS),* Depressive symptoms *(MADRS),* Functioning *(GAF)* | 3 months; median of 6.7 years |
| Bowie, 2012 | USA | Antidepressants and antipsychotics versus off medication and healthy control | n/a | Antidepressant group: n=15, age= 15.52 (1.94), Antipsychotic: n=11, age=16.44 (1.52)  Off medication: n=27, age= 16.40 (2.0)  HC: n=17, age= 16.41 (2.26) | 70% | Prodromal psychotic symptoms *(SIPS),* Depressive symptoms *(BDI),* Anxiety symptoms *(BAI),* Verbal learning *(CVLT),* Visual motor speed and conceptual shifting *(parts A and B of trail making task),* working memory *(letter-number span test),* sustained attention *(continuous performance test)* | Treated pharmacologically for at least 3 months before follow up; 6 months |
| Brazzale, 2018 | Italy | Video therapy for identity and self-esteem | Metacognitive treatment: Enhancing interpersonal skills, improve theory of mind, self-serving attribution bias, improving insight,  Videotherapy: improve self-perception of identity | n=18, age= 17.6 (3.02)  No control | 44.4% | Psychological and behavioural disturbance *(YSR),* Mentalising and social abilities *(SIB, TOMT),* Visual perception control *(TVIC)* | 4 x 1hour 20 sessions for 4 weeks |
| Cornblatt, 2007 | USA | Antidepressants versus Antipsychotics | n/a | Antidepressant group n=20, age= 16.3 (2.6)  Antipsychotic group: n=28, age= 15.7 (1.9) | 60.4% | Conversion to psychosis (SOPS), Prodromal symptoms (SOPS), Medication adherence (parent, participant and clinican reports) | Treated pharmacologically for at least 8 weeks; every 6 months for up to 5 years |
| Grano, 2016 | Finland | Family and community integrated treatment model (FCTM) versus treatment as usual | Family therapy (to reduce internal conflicts, educate families), open diaglogue and CBT (normalising and psychoeducation) and community approaches to reduce stress factors | FCTM group: n=28, age= 15.5 (1.6)  TAU group: n=28, age= 16.3 (0.8) | 32% | Prodromal psychotic symptoms (SIPS), Depressive symptoms (BDI), Anxiety (BAI), Hopelessness (BHI), Functioning (GAF) | 12 months; 12 months |
| Holzer, 2012 | Switzerland | Computer aided cognitive remediation for CHR-P and with psychotic disorders versus computer games | Remediate attention, improve working memory, coordination, reasoning, self-esteem and self- control. Also focus on perceptual discrimination, processing speed, response inhibition. | CACR group: n=18, age= 15.4 (1.3)  computer games group: n=14, age= 15.7 (1.4) | 57% | Positive and negative psychotic symptoms (PANSS), Functioning (SOFAS, social and occupational; HoNOSCA), Cognitive change (RBANS) | 8 weeks; 9th week |
| Janssen, 2021 | The Netherlands | CBT alone versus CBT with additional psychomotor and family therapy for CHR-P and help-seeking population | Focussing on abnormal movements or posturing, relaxation techniques, exercise. Family therapy to improve family interactions | CHR-P group: n=61, age=15.8 (3.2)  HSP: n=82, 17.1 (3.2) | 47.5% | Attenuated psychotic symptoms (CAARMS), Functioning (GAF and HoNOSCA) | 5 months; 5 months |
| McAusland, 2018 | Canada | Heartrate biofeedback | Paced breathing, focus on control over bodily sensations to soothe distress. | n=20, age= 16.8 (2.4)  No control group | 33% | Prodromal symptoms (COPS, SIPS), Anxiety symptoms (Zung SAS), Distress (K10), Social interaction anxiety Scale (SIAS), Functioning (GF:R and GF:S, social and role functioning scales), | 4 weeks; 4 and 8 weeks |
| McFarlane, 2015 | USA | Family aided assertive community treatment (FACT) vs community care (CC) for clinically lower risk | Psychoeducational family groups, supported education and employment, holistic approach to support management of illness but also social stressor management | FACT: n=205, age= 16.40 (3.30)  CC: n=87, age= 16.23 (3.18) | 63.5% | Transition to psychosis (POPS) Prodromal psychosis symptoms (SIPS) Functioning (GAF, GF:R and GF:S), Quality of life: interpersonal and intrapsychic (Heinrich's QLS) | 6 months; 6, 12, 24 months |
| Miklowitz, 2014 | USA | Family focussed therapy versus enhanced care (psychoeducation) | Psychoeducation summarising stressors and coping strategies, communication, problem solving within the family | FFT: n=66, age= 17.3 (4.20)  EC: n=63, age= 17.4 (3.9) | 57.8% | Conversion to psychosis (SIPS/SOPS) Prodromal psychotic symptoms (SOPS) Functioning (GAF, GF:R and GF:S, role and social) | 6 months; 6 months |
| Mossaheb, 2013 | Austria | Omega-3 PUFA vs coconut oil placebo | N/A | Omega-3: n=40, age= 16.8 (2.4)  Placebo: n=41, age= 16.0 (1.7) | 33% | Conversion to psychosis (PANSS severity thresholds), Positive and negative psychotic symptoms (PANSS), Depressive symptoms (MADRS), Functioning (GAF), Adverse effects (UKU) | 3 months; 3, 6, 12 months |
| O'Brien, 2014 | USA | Family focussed therapy versus enhanced care (psychoeducation) | Psychoeducation summarising stressors and coping strategies, communication, problem solving within the family | FFT: n=38, age= 17.2 (4.24)  EC: n=28, age= 16.5 (2.50) | 56.2% | Family interactional behaviour during a problem solving task/ family behaviour observation assessment | 6 months; 6 months |
| O'Brien, 2007 | USA | Psychoeducational multi-family group versus declined participation | Psychoeducation about the prodromal state, biological explanation of symptoms, recommendations for creating positive environment for the individual. Problem solving within family. | PMFG: n=16, age= 15.7  Declined participation: n=13, age=16.1 | 59.6% | Conversion to psychosis (SIPS), Prodromal psychosis symptoms (SIPS/SOPS), Global functioning (GAF), Family adaptability and cohesion, Coping | 9 months; 9 months |
| O'Brien Cannon, 2022 | USA | Family Focused Therapy for CHR-P versus enhance care (psychoeducation) with comorbid anxiety disorder or no anxiety disorder | Psychoeducation, identifying stressors of symptoms, coping strategies. Communication enhancement, focus on active listening. Problem solving. | Anxiety FFT group: n=32, age=17.1 (3.70). Non-anxiety FFT group: n=34, age= 17.6 (4.40)  Anxiety EC group: n=35, non-anxiety EC group: n=28 (no breakdown of age for treatment group) | 58.60% | Anxiety symptoms (Zung SRAS) Family communication (interaction was coded for affection, compliments, listening behaviour, calm speaking, irritability, anger, complaints, criticism and off-task comments) | 6 months; 6, 12 months |
| Pitzianti 2019 | Italy | Risperidone antipsychotic versus drug naïve CHR-P and healthy controls | N/A | Riseridone group: n=15, age= 15.2 (1.7)  Drug naïve: n=15, age=14.6 (2.20). HC: n=25, age=14.4 (3.40) | 65.7% | PANESS (physical and neurological assessment of subtle signs, motor functioning) | 6 months; 6 months |
| Stain, 2016 | Australia | CBT versus non-directive relfective listening (NDRL) | Problem-solving based, behavioural experiments, normalising, generating and evaluating alternative beliefs. | CBT group: n=30, age=16.2 (2.73)  NDRL group: n=27, age= 16.5 (3.16) | 40.5% | Transition to psychosis (CAARMS and SCID or K-SADS), Subclinical psychotic symtpoms (CAARMS), Depression and anxiety (BSI), Functioning (GAF), quality of life: interpersonal and intrapsychic (Heinrich's QLS) | 6 months; 6, 12 months |
| Urben, 2012 | Switzerland | Computer aided cognitive remediation for CHR-P and with psychotic disorders versus computer games | Cognitive training to remediate attention, improve working memory, coordination, reasoning, self-esteem and self- control. Also focus on perceptual discrimination, processing speed, response inhibition. | CACR group: n=12, 16.6% CHR-P, age= 15.2 (1.27)  Computer games group: n=10, 40% CHR-P, age= 16 (1.25) | 64.2% | Processing speed (WAIS-III and WISC-IV), Working Memory (WAIS-III and WISC-IV), Reasoning (WAIS-III and WISC-IV), Executive functioning (Colour stroop task), Symptoms (CGI) | 8 weeks; 6 months |
| Waite, 2023 | UK | SleepWell therapy versus usual care (UC) | Increasing the need for sleep by increasing daytime activity, timing of sleep by realigning sleep patterns with environment light/dark cues and re-establish circadian rhythms also re-establishing bed-sleep association. Also worry reduction, nighttime relaxation and cognitive restructuring. | Sleepwell: n=21, age= 17.0 (2.2),  UC: n=19, age= 16.8 (2.8) | 48% | Psychotic experiences (CAARMS) and hallucinatory experiences (specific psychotic experiences questionnaire- SPEQ-H), paranoid thoughts (paranoid thoughts scale- R-GPTS), depression (depression anxiety stress scales- DASS-21), social functioning (work and social adjustment scale). | 8 hours over 12 weeks then 3 month and 9 months follow ups. |
| Woodberry, 2020 | USA | Computer Aided Learning for managing stress with parents (heartrate biofeedback) | Aim of emotion regulation and calming interpersonal interactions to keep heart rate low. Facilitate stress-management mastery. Psychoeducation about stress responses and stressors. | n=11, age= 17.2 (2.30)  No control group | 73% | Prodromal symptoms (SIPS), Functioning (GAF; GF:R and GF:S, role and social), Perceived stress, criticism and warmth (PSS, PCS and PWS), Conflict behaviour (CBQ) | 12 weeks; 12 weeks |
| Woods, 2007 | USA | Aripiprazole antipsychotic | n/a | n=14, age= 17.1 (5.50)  No control group | 53% | Prodromal psychotic symptoms (SOPS)  Depression symptoms (CDRS)  Manic symptoms (YMRS) Anxiety symptoms (BAI) Functioning (GAF, GF:R and GF:S, role and social) Quality of life (Heinrich-Carpenter QoL role functioning sub-scale) Processing speed (Stroop colour task) Working memory (letter-number sequencing) Executive functioning (WCST) Safety (SAFTEE) | 8 weeks; 8 weeks |
| Woods, 2013a | USA | Glycine versus sucrose placebo | n/a | Glycine group: n=4, age= 15.3 (0.50)  Sucrose group: n=4, age= 16.5 (2.40) | 75% | Prodromal psychotic symptoms (SOPS)  Depressive symptoms (MADRS) Functioning (GAF) | 8 weeks; 8 weeks |
| Woods, 2013b | USA | Glycine | n/a | n=10, age= 17.3 (3.30)  No control group | 70% | Prodromal psychotic symptoms (SOPS)  Depressive symptoms (MADRS) Functioning (GAF) | 8 weeks; 8 weeks |

**Abbreviations in order of appearance.** Study design column: RCT= Randomised Controlled Trials, NRSI= Non-Randomised Studies of Intervention. Intervention and control column: FFT= Family Focused Therapy, EC= Enhanced Care, FCTM= Family and Community Oriented Integrative Treatment Model, TAU= Treatment as Usual, FACT= Family-Aided Assertive Community Treatment, EFEP= Early First Episode Psychosis, PMFG= Psycho-educational Multi-Family Group, CACR= Computer-Aided Cognitive Remediation, PMT= Psychomotor Therapy, FT=Family Therapy, HSP= Help-Seeking Population, NDRL= Non-Directive Reflective Listening, BPD= Borderline Personality Disorder, PUFA= Polyunsaturated Fatty Acid, HC= Healthy Controls. Instrument to assess CHR-P column: SIPS= Structured Interview for Psychosis-Risk Syndromes, PROD= Screen for Prodromal Symptoms of Psychosis, SOPS= Scale of Prodromal Symptoms, COPS= Criteria of Prodromal Symptoms, CAARMS= Comprehensive Assessment of At Risk Mental States, ERIraos= Early Recognition Inventory for the Retrospective Assessment of Onset of Schizophrenia, PANSS= Positive And Negative Syndrome Scale. Outcome column: SAS= Self-rated Anxiety Scale, BDI= Beck’s Depression Inventory, BAI= Beck’s Anxiety Inventory, BHI= Beck’s Hopelessness Inventory, GAF= Global Assessment of Functioning, GF:R= Assessment of role Functioning, GF: S= Assessment of Social Functioning, POPS= Presence of Psychosis Scale, PSS= Perceived Stress Scale, CBQ= Conflictual Behaviour Questionnaire, SOFAS= Social and Occupational Functioning Assessment Scale, HoNOSCA= Health of the Nation Outcome Scales for Children and Adolescents, RBANS= Repeatable Battery for Assessment of Neurological Status, WAIS= Wechsler Adult Intelligence Scales, WISC= Wechsler Intelligence Scale for Children, CGI= Clinical Global Improvement, SCID= Structured Clinical Interview for DSM-5 Disorders, K-SADS= Kiddie Schedule for Affective Disorders and Schizophrenia, YSR=Youth Self-Report, SIB= Social Intelligence Battery, VVIQ= Vividness of Visual Imagery Questionnaire ,VMIQ= Vividness of Moving Images Questionnaire, K10= Kessler Psychological Distress Scale, SIAS= Social interaction Anxiety Scale, MADRS= Montgomery-Asperg Depression Rating Scale, CPT= Continuous Performance Task, CVLT= California Verbal learning Test, PANASS= Physiological and Neurological Assessment of Subtle Signs, CDRS= Children’s Depression Rating Scale, YMRS= Youth Mania Rating Scale.

**
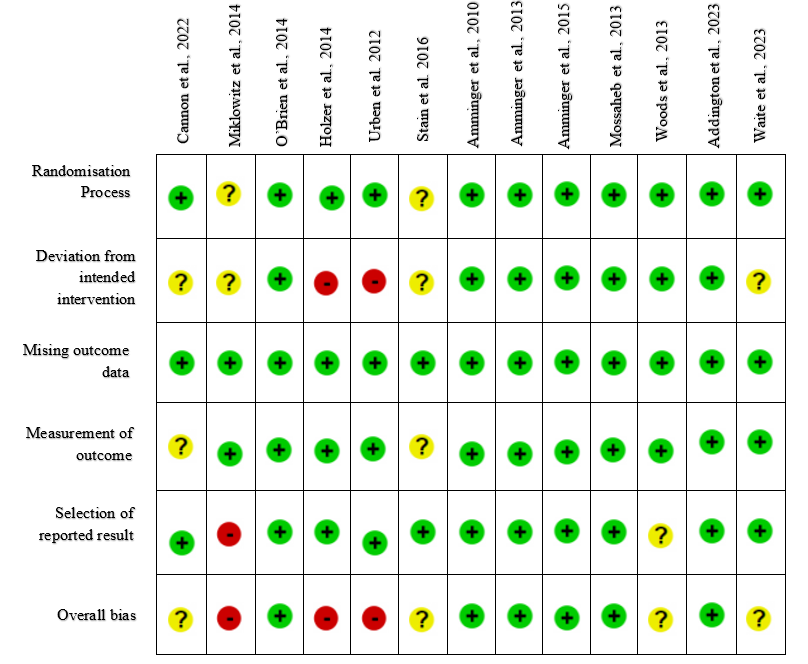
****Figure S1.** ROB-2 quality assessment results.

| **Author and year** | **Baseline confounding** | **Selection of participants** | **Classification of intervention** | **Deviation from intended interventions** | **Missing data** | **Measurement of outcomes** | **Selection of reported results** | **Overall risk of bias** |
| --- | --- | --- | --- | --- | --- | --- | --- | --- |
| Grano et al., 2016 | Moderate | Serious | Low | Moderate | Low | Low | Low | Serious |
| McFarlane et al., 2015 | Low | Serious | Low | Moderate | Moderate | Low | Moderate | Serious |
| O’Brien et al., 2007 | Low | Low | Low | Low | Moderate | Moderate | Moderate | Moderate |
| Woodberry et al., 2020 | Moderate | Low | Low | Low | Low | Moderate | Low | Moderate |
| Janssen et al., 2021 | Moderate | Low | Low | Low | Low | Low | Low | Moderate |
| Brazzale et al., 2018 | NI | Low | Low | Low | NI | Moderate | Low | Moderate |
| McAusland et al., 2016 | Moderate | Low | Low | Moderate | Low | Low | Low | Moderate |
| Bowie et al., 2012 | Low | Low | Moderate | Low | NI | Moderate | Low | Moderate |
| Cornblatt et al., 2007 | Moderate | Serious | Moderate | Low | Moderate | Moderate | Low | Serious |
| Pitzianti et al., 2019 | Low | Low | Low | Low | NI | Moderate | Low | Moderate |
| Woods et al., 2007 | Low | Low | Low | Low | Low | Moderate | Moderate | Moderate |
| Woods et al., 2013 | Low | Low | Low | Low | Low | Moderate | Low | Moderate |

**Table S5.** ROBINS-I quality assessment results.

**Table S6.** ROB2 assessment for all included studies for each outcome.

| **Author and year** | **Randomisation process** | | **Deviations from intended interventions** | | | **Missing outcome data** | **Measurement of outcomes** | | **Selection of reported results** | | **Overall risk of bias** |
| --- | --- | --- | --- | --- | --- | --- | --- | --- | --- | --- | --- |
| **Transition to psychosis** | | | | | | | | | | | |
| Addingotn et al., 2023 | Low risk | | Low risk | | | Low risk | Low risk | | Low risk | | Low risk |
| Amminger et al., 2010 | Low risk | | Low risk | | | Low risk | Low risk | | Low risk | | Low risk |
| Amminger et al., 2015 | Low risk | | Low risk | | | Low risk | Low risk | | Low risk | | Low risk |
| Cornblatt et al., 2007 | High risk | | Low risk | | | Some concerns | Some concerns | | Low risk | | High risk |
| McFarlane et al., 2015 | High risk | | Some concerns | | | Some concerns | Low risk | | Some concerns | | High risk |
| Miklowtiz et al., 2014 | Some concerns | | Some concerns | | | Low risk | Low risk | | High risk | | High risk |
| Mossaheb et al., 2013 | Low risk | | Low risk | | | Low risk | Low risk | | Low risk | | Low risk |
| O’Brien et al., 2007 | High risk | | Low risk | | | Some concerns | Some concerns | | Some concerns | | High risk |
| Stain et al., 2016 | Some concerns | | Some concerns | | | Low risk | Some concerns | | Low risk | | Some concerns |
| **Positive symptoms** | | | | | | | | | | | |
| Addingotn et al., 2023 | | Low risk | | Low risk | Low risk | | Low risk | Low risk | | Low risk | |
| Amminger et al., 2010 | | Low risk | | Low risk | Low risk | | Low risk | Low risk | | Low risk | |
| Amminger et al., 2015 | | Low risk | | Low risk | Low risk | | Low risk | Low risk | | Low risk | |
| Bowie et al., 2012 | | High risk | | Low risk | Some concerns | | Some concerns | Low risk | | High risk | |
| Cornblatt et al., 2007 | | High risk | | Low risk | Some concerns | | Some concerns | Low risk | | High risk | |
| Grano et al., 2016 | | High risk | | Some concerns | Low risk | | Low risk | Low risk | | High risk | |
| Holzer et al., 2012 | | Low risk | | High risk | Low risk | | Low risk | Low risk | | High risk | |
| Janssen et al., 2021 | | High risk | | Low risk | Low risk | | Low risk | Low risk | | High risk | |
| McAusland et al., 2018 | | High risk | | Some concerns | Low risk | | Low risk | Low risk | | High risk | |
| McFarlane et al., 2015 | | High risk | | Some concerns | Some concerns | | Low risk | Some concerns | | High risk | |
| Miklowtiz et al., 2014 | | Some concerns | | Some concerns | Low risk | | Low risk | High risk | | High risk | |
| Mossaheb et al., 2013 | | Low risk | | Low risk | Low risk | | Low risk | Low risk | | Low risk | |
| O’Brien et al., 2007 | | High risk | | Low risk | Some concerns | | Some concerns | Some concerns | | High risk | |
| Stain et al., 2016 | | Some concerns | | Some concerns | Low risk | | Some concerns | Low risk | | Some concerns | |
| **Author and year** | | **Randomisation process** | | **Deviations from intended interventions** | **Missing outcome data** | | **Measurement of outcomes** | **Selection of reported results** | | **Overall risk of bias** | |
| **Positive symtpoms cont.** | | | | | | | | | | | |
| Woodberry et al., 2021 | | High risk | | Low risk | Low risk | | Some concerns | Low risk | | High risk | |
| Woods et al., 2007 | | High risk | | Low risk | Low risk | | Some concerns | Some concerns | | High risk | |
| Woods et al., 2013a | | Low risk | | Low risk | Low risk | | Low risk | Some concerns | | Some concerns | |
| Woods et al., 2013b | | High risk | | Low risk | Low risk | | Some concerns | Low risk | | High risk | |
| **Negative symptoms** | | | | | | | | | | | |
| Addingotn et al., 2023 | | Low risk | | Low risk | Low risk | | Low risk | Low risk | | Low risk | |
| Amminger et al., 2010 | | Low risk | | Low risk | Low risk | | Low risk | Low risk | | Low risk | |
| Amminger et al., 2015 | | Low risk | | Low risk | Low risk | | Low risk | Low risk | | Low risk | |
| Bowie et al., 2012 | | High risk | | Low risk | Some concerns | | Some concerns | Low risk | | High risk | |
| Cornblatt et al., 2007 | | High risk | | Low risk | Some concerns | | Some concerns | Low risk | | High risk | |
| Grano et al., 2016 | | High risk | | Some concerns | Low risk | | Low risk | Low risk | | High risk | |
| Holzer et al., 2012 | | Low risk | | High risk | Low risk | | Low risk | Low risk | | High risk | |
| McAusland et al., 2018 | | High risk | | Some concerns | Low risk | | Low risk | Low risk | | High risk | |
| McFarlane et al., 2015 | | High risk | | Some concerns | Some concerns | | Low risk | Some concerns | | High risk | |
| Miklowtiz et al., 2014 | | Some concerns | | Some concerns | Low risk | | Low risk | High risk | | High risk | |
| Mossaheb et al., 2013 | | Low risk | | Low risk | Low risk | | Low risk | Low risk | | Low risk | |
| O’Brien et al., 2007 | | High risk | | Low risk | Some concerns | | Some concerns | Some concerns | | High risk | |
| Stain et al., 2016 | | Some concerns | | Some concerns | Low risk | | Some concerns | Low risk | | Some concerns | |
| Woodberry et al., 2021 | | High risk | | Low risk | Low risk | | Some concerns | Low risk | | High risk | |
| Woods et al., 2007 | | High risk | | Low risk | Low risk | | Some concerns | Some concerns | | High risk | |
| Woods et al., 2013a | | Low risk | | Low risk | Low risk | | Low risk | Some concerns | | Some concerns | |
| Woods et al., 2013b | | High risk | | Low risk | Low risk | | Some concerns | Low risk | | High risk | |
| **Total symptoms** | |  | |  |  | |  |  | |  | |
| Amminger et al., 2010 | | Low risk | | Low risk | Low risk | | Low risk | Low risk | | Low risk | |
| Amminger et al., 2015 | | Low risk | | Low risk | Low risk | | Low risk | Low risk | | Low risk | |
| Bowie et al., 2012 | | High risk | | Low risk | Some concerns | | Some concerns | Low risk | | High risk | |
| Cornblatt et al., 2007 | | High risk | | Low risk | Some concerns | | Some concerns | Low risk | | High risk | |
| **Author and year** | | **Randomisation process** | | **Deviations from intended interventions** | **Missing outcome data** | | **Measurement of outcomes** | **Selection of reported results** | | **Overall risk of bias** | |
| **Total symptoms cont.** | | | | | | | | | | | |
| Grano et al., 2016 | | High risk | | Some concerns | Low risk | | Low risk | Low risk | | High risk | |
| McAusland et al., 2018 | | High risk | | Some concerns | Low risk | | Low risk | Low risk | | High risk | |
| McFarlane et al., 2015 | | High risk | | Some concerns | Some concerns | | Low risk | Some concerns | | High risk | |
| Miklowtiz et al., 2014 | | Some concerns | | Some concerns | Low risk | | Low risk | High risk | | High risk | |
| Mossaheb et al., 2013 | | Low risk | | Low risk | Low risk | | Low risk | Low risk | | Low risk | |
| O’Brien et al., 2007 | | High risk | | Low risk | Some concerns | | Some concerns | Some concerns | | High risk | |
| Stain et al., 2016 | | Some concerns | | Some concerns | Low risk | | Some concerns | Low risk | | Some concerns | |
| Waite et al., 2023 | | Low risk | | Some concerns | Low risk | | Low risk | Low risk | | Some concerns | |
| Woodberry et al., 2021 | | High risk | | Low risk | Low risk | | Some concerns | Low risk | | High risk | |
| Woods et al., 2007 | | High risk | | Low risk | Low risk | | Some concerns | Some concerns | | High risk | |
| Woods et al., 2013a | | Low risk | | Low risk | Low risk | | Low risk | Some concerns | | Some concerns | |
| Woods et al., 2013b | | High risk | | Low risk | Low risk | | Some concerns | Low risk | | Some concerns | |
| **Depression** | | | | | | | | | | | |
| Addingotn et al., 2023 | | Low risk | | Low risk | Low risk | | Low risk | Low risk | | Low risk | |
| Amminger et al., 2010 | | Low risk | | Low risk | Low risk | | Low risk | Low risk | | Low risk | |
| Amminger et al., 2015 | | Low risk | | Low risk | Low risk | | Low risk | Low risk | | Low risk | |
| Bowie et al., 2012 | | High risk | | Low risk | Some concerns | | Some concerns | Low risk | | High risk | |
| Grano et al., 2016 | | High risk | | Some concerns | Low risk | | Low risk | Low risk | | High risk | |
| Mossaheb et al., 2013 | | Low risk | | Low risk | Low risk | | Low risk | Low risk | | Low risk | |
| Stain et al., 2016 | | Some concerns | | Some concerns | Low risk | | Some concerns | Low risk | | Some concerns | |
| Waite et al., 2023 | | Low risk | | Some concerns | Low risk | | Low risk | Low risk | | Some concerns | |
| Woods et al., 2007 | | High risk | | Low risk | Low risk | | Some concerns | Some concerns | | High risk | |
| Woods et al., 2013a | | Low risk | | Low risk | Low risk | | Low risk | Some concerns | | Some concerns | |
| Woods et al., 2013b | | High risk | | Low risk | Low risk | | Some concerns | Low risk | | Some concerns | |
| **Functioning** | | | | | | | | | | | |
| Addingotn et al., 2023 | | Low risk | | Low risk | Low risk | | Low risk | Low risk | | Low risk | |
| Amminger et al., 2010 | | Low risk | | Low risk | Low risk | | Low risk | Low risk | | Low risk | |
| **Author and year** | | **Randomisation process** | | **Deviations from intended interventions** | **Missing outcome data** | | **Measurement of outcomes** | **Selection of reported results** | | **Overall risk of bias** | |
| **Functioning cont.** | | | | | | | | | | | |
| Amminger et al., 2015 | | Low risk | | Low risk | Low risk | | Low risk | Low risk | | Low risk | |
| Grano et al., 2016 | | High risk | | Some concerns | Low risk | | Low risk | Low risk | | High risk | |
| Holzer et al., 2012 | | Low risk | | High risk | Low risk | | Low risk | Low risk | | High risk | |
| Janssen et al., 2021 | | High risk | | Low risk | Low risk | | Low risk | Low risk | | High risk | |
| McAusland et al., 2018 | | High risk | | Some concerns | Low risk | | Low risk | Low risk | | High risk | |
| McFarlane et al., 2015 | | High risk | | Some concerns | Some concerns | | Low risk | Some concerns | | High risk | |
| Miklowtiz et al., 2014 | | Some concerns | | Some concerns | Low risk | | Low risk | High risk | | High risk | |
| Mossaheb et al., 2013 | | Low risk | | Low risk | Low risk | | Low risk | Low risk | | Low risk | |
| O’Brien et al., 2007 | | High risk | | Low risk | Some concerns | | Some concerns | Some concerns | | High risk | |
| Stain et al., 2016 | | Some concerns | | Some concerns | Low risk | | Some concerns | Low risk | | Some concerns | |
| Waite et al., 2023 | | Low risk | | Some concerns | Low risk | | Low risk | Low risk | | Some concerns | |
| Woodberry et al., 2021 | | High risk | | Low risk | Low risk | | Some concerns | Low risk | | High risk | |
| Woods et al., 2007 | | High risk | | Low risk | Low risk | | Some concerns | Some concerns | | High risk | |
| Woods et al., 2013a | | Low risk | | Low risk | Low risk | | Low risk | Some concerns | | Some concerns | |
| Woods et al., 2013b | | High risk | | Low risk | Low risk | | Some concerns | Low risk | | Some concerns | |

**Table S7.** Results of heterogeneity analysis and random effects model.

| **Outcome** | **No. of**  **Studies** | **N**  **INT** | **N CTRL** | **Odds ratio** | | | | **z Score** | | **P** | | **Test for Heterogeneity** | | | | **Prediction Interval** | | |
| --- | --- | --- | --- | --- | --- | --- | --- | --- | --- | --- | --- | --- | --- | --- | --- | --- | --- | --- |
|  |  |  |  | **Odds ratio** | **95 CI** | |  | |  | | **Q** | | **I^2^** | **P** |  | |  |  |
| Transition to psychosis | 3 | 262 | 140 | 0.711 | 0.149 | 3.395 | -0.427 | | 0.669 | | 8.694 | | 76.996 | 0.013 | 1.208 | | 1.459 |  |
| **Outcome** | **No. of studies** | **N INT** | **N CTRL** | **Hedge’s g** | |  | **z Score** | | **P** | | **Test for Heterogeneity** | | | | **Prediction Interval** | | |  |
|  |  |  |  | **Mean** | **95 CI** | |  | |  | | **Q** | | **I^2^** | **P** |  | |  |  |
| Positive symptoms | 3 | 249 | 131 | 0.379 | 0.055 | 0.703 | 2.294 | | 0.022 | | 2.910 | | 31.266 | 0.233 | TAU  0.169 | | TAU2  0.028 |  |
| Negative symptoms | 3 | 249 | 131 | 0.583 | 0.187 | 0.980 | 2.882 | | 0.004 | | 0.318 | | 0.000 | 0.853 | 0.000 | | 0.000 |  |
| Total symptoms | 2 | 45 | 44 | 0.677 | 0.249 | 1.105 | 3.102 | | 0.002 | | 0.422 | | 0.000 | 0.517 | Tau 0.000 | | Tau 2  0.000 |  |
| Depressive symptoms | 2 | 45 | 44 | 0.936 | -0.788 | 2.661 | 1.064 | | 0.287 | | 4.022 | | 75.137 | 0.045 | Tau 1.105 | | Tau 2  1.222 |  |
| Functioning | 4 | 304 | 182 | 0.944 | 0.052 | 1.836 | 2.075 | | 0.038 | | 49.598 | | 93.951 | 0.000 | Tau 0.879 | | TAU2 0.773 |  |

**Figure S2.** Forest plot for depression symptoms outcome.


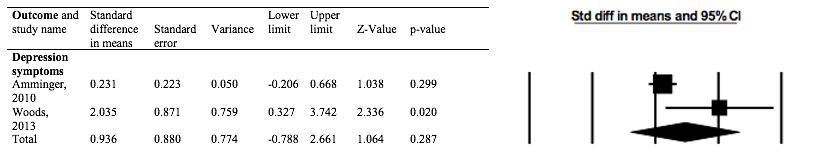


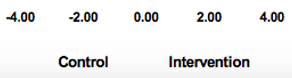


**Table S8.** Publication bias: Egger’s test results.

|  | **Intercept** | **SE** | **95% CI** | | **T value** | **P-value** |
| --- | --- | --- | --- | --- | --- | --- |
| **Transition to psychosis** | -6.152 | 9.537 | -127.3 | 115.03 | 0.645 | 0.635 |
| **Negative symptoms** | -0.662 | 0.266 | -4.038 | 2.714 | 2.491 | 0.243 |
| **Total symptoms** | N/A | N/A | N/A | N/A | N/A | **N/A** |
| **Depressive symptoms** | N/A | N/A | N/A | N/A | N/A | N/A |
| **Functioning** | -13.865 | 2.489 | -24.576 | -3.154 | 5.57 | 0.0307 |

**Figure S3.** Funnel plot for Transition to psychosis outcome.

**Figure S4.** Funnel plot for positive symptoms outcome.

**Figure S5**. Funnel plot for negative symptoms outcome.

**Figure S6**. Funnel plot for global functioning outcome.

**Results S1.** Narrative synthesis and discussion of findings for secondary outcomes.

*Disorganised symptoms*

6 studies reported on disorganised symptoms (9, 13, 16, 19, 23, 24). The interventions that produced a change in disorganised symptoms over time were pharmacological including aripiprazole medication and glycine. Of these interventions, the largest effect size was produced by glycine (d=-1.05), however, this significant change was only seen after 12 weeks of receiving glycine. Across any of the eligible studies, there were no group differences between the interventions and controls in change in disorganised symptoms. The only two interventions that improved disorganised symptoms over time were aripiprazole and glycine with glycine producing a large effect size but again, this may be explained by the small sample size in this study. These two studies were naturalistic and hence further research into whether aripiprazole and 12 weeks or more of glycine would outperform controls of usual care or best current practice conditions is needed before confirming that these interventions are effective.

*Anxiety symptoms or distress related to psychotic symptoms:*

8 studies reported on anxiety (3, 6, 7, 9, 12, 19, 22, 23) however there was too much heterogeneity in the scales used to assess anxiety, control groups or samples across studies to include this outcome in the meta-analysis. The interventions that produced a significant change were pharmacological (omega-3 PUFA and aripiprazole) or had a cognitive-behavioural basis (CBT, metacognitive techniques and biofeedback). Reported effect sizes were small to medium. The interventions that outperformed the control groups in anxiety reduction were FFT and NDRL. Both these interventions share a focus on active listening and problem solving of specific issues salient to the family or individual- an element lacking in the controls. In adult samples, psychological interventions including family therapy and CBT were ineffective at improving symptom-related distress (Mei et al., 2021). Similarly, CBT was ineffective compared to NDRL at reducing symptom related anxiety in children and adolescents. Family therapy may however be more impactful for those under 18 who are more likely to share a living environment with their family than adults.

*Family Interactional behaviour*

4 studies reported on family interactional behaviour (7, 16, 17, 20) and all studies that reported significant improvement had an emphasis on using family support to reduce the impact of symptoms and enhance communication to moderate the effects of conflictual interactions. However, no interventions outperformed the control groups and this may be because of the shared element of psychoeducation between the controls and interventions and its impact on family interactions over elements in the intervention group alone such as communication enhancement. This psychoeducation element encourages family members to become more knowledgeable about CHR-P symptoms and experiences, facilitating deeper understanding of behaviours which may improve empathetic reasoning during family conflicts (Gearing, 2008).

*Cognition*

6 studies reported on cognitive outcomes (5, 6, 10, 20, 22, 23) however there was much heterogeneity in the scales and paradigms used to assess these. Antipsychotic medications were seen to worsen verbal memory and sustained attention from baseline across two separate studies (3,20) whereas antidepressant medications were seen to improve these outcomes. Glycine improved both verbal memory and processing speed but only did so after 12 weeks and not at 8 weeks. CACR improved working memory, reasoning abilities and visual perception from baseline however, a similar computer-aided videotherapy failed to improve visual perception from baseline.

Two interventions outperformed control groups: CACR and antidepressant medication but the effects of CACR over the control group at improving visual perception did not last as seen in a follow up assessment 6 month later (18).

Antidepressants outperformed the off medication control group and the antipsychotics group at improving sustained attention and verbal memory from baseline.

The only psychological therapy that improved cognitive abilities, executive functioning and visual perception was CACR however a similar computer assisted metacognitive intervention did not produce any change in these outcomes. Therefore the effective element of this intervention may be the cognitive remediation and this was supported by a systematic review on cognitive remediation for older CHR-P samples (Glenthøj et al., 2017).

*Motor functioning*

Two studies reported on motor functioning (18, 23) and both looked at the effects of second generation antipsychotics. There was no change in involuntary movements after 8 weeks receiving aripiprazole and similarly, even after 6 months of taking risperidone there were no differences in involuntary movements compared to those who were drug-naïve. Little research has been conducted on interventions that target motor abnormalities for CHR-P individuals and this may be an important avenue of future research given that baseline involuntary movements can predict transition to psychosis years later (Callaway et al., 2014).

References:

ADDINGTON, J., LIU, L., BRAUN, A., BRUMMITT, K., CADENHEAD, K. S., CORNBLATT, B. A., HOLDEN, J. L., GRANHOLM, E. 2023. Cognitive-Behavioural Social Skills Training: Outcome of a Randomized Controlled Trial for Youth at Risk of Psychosis. *Schizophrenia Bulletin Open*. doi:10.1093/schizbullopen/sgad020.

AMMINGER, G. P., CHANEN, A. M., OHMANN, S., KLIER, C. M., MOSSAHEB, N., BECHDOLF, A., NELSON, B., THOMPSON, A., MCGORRY, P. D., YUNG, A. R. & SCHAFER, M. R. 2013. Omega-3 Fatty Acid Supplementation in Adolescents With Borderline Personality Disorder and Ultra-High Risk Criteria for Psychosis: A Post Hoc Subgroup Analysis of a Double-Blind, Randomized Controlled Trial. *Canadian Journal of Psychiatry-Revue Canadienne De Psychiatrie,* 58**,** 402-408.

AMMINGER, G. P., SCHAFER, M. R., PAPAGEORGIOU, K., KLIER, C. M., COTTON, S. M., HARRIGAN, S. M., MACKINNON, A., MCGORRY, P. D. & BERGER, G. E. 2010. Long-Chain omega-3 Fatty Acids for Indicated Prevention of Psychotic Disorders A Randomized, Placebo-Controlled Trial. *Archives of General Psychiatry,* 67**,** 146-154.

AMMINGER, G. P., SCHÄFER, M. R., SCHLÖGELHOFER, M., KLIER, C. M. & MCGORRY, P. D. 2015. Longer term outcome in the prevention of psychotic disorders by the Venna omega-3 study. *Nature Archives,* 6 (7934), pp.1-7.

ANDREASEN, N. C. 1989. The Scale for the Assessment of Negative Symptoms (SANS): Conceptual and Theoretical Foundations. *British Journal of Psychiatry*, 155(S7):49-52. doi:10.1192/S0007125000291496

BOWIE, C. R., MCLAUGHLIN, D., CARRION, R. E., AUTHER, A. M. & CORNBLATT, B. A. 2012. Cognitive changes following antidepressant or antipsychotic treatment in adolescents at clinical risk for psychosis. *Schizophrenia Research,* 137**,** 110-117.

BRAZZALE, R., MADDALENA, Y., COZZI, A. & BRAZZALE, L. 2018. New Pathways of Intervention for Adolescents at Clinical High Risk of Psychosis: Improving Meta-representation Skills and Strengthening Identity with Video-Confrontation Techniques. *Adolescent Psychiatry,* 8**,** 121-132.

CALLAWAY, D. A., PERKINS, D. O., WOODS, S. W., LIU, L., ADDINGTON, J. 2014. Movement abnormalities predict transitioning to psychosis in individuals at clinical high risk for psychosis, *Schizophrenia Research*, 159 (2–3), 263-266

CORNBLATT, B. A., LENCZ, T., SMITH, C. W., OLSEN, R., AUTHER, A. M., NAKAYAMA, E., LESSER, M. L., TAI, J. Y., SHAH, M. R., FOLEY, C. A., KANE, J. M. & CORRELL, C. U. 2007. Can antidepressants be used to treat the schizophrenia prodrome? Results of a prospective, naturalistic treatment study of adolescents. *Journal of Clinical Psychiatry,* 68**,** 546-557.

FUX, L., WALGER, P., SCHIMMELMANN, B., SCHULTZE-LUTTER, F. (2013). The Schizophrenia Proneness Instrument, Child and Youth version (SPI-CY): Practicability and discriminative validity. *Schizophrenia research*. 146. 10.1016/j.schres.2013.02.014.

GEARING, R. E. 2008. Evidence-based family psychoeducational interventions for children and adolescents with psychotic disorders. *Journal Canadian Academic Child and Adolescent Psychiatry.* 17(1):2-11.

GLENTHØJ, L.B., FAGERLUND, B., BAK, N., HJORTHØJ, C., GREGERSEN, M., KRISTENSEN, T.D., WENNEBERG, C., KRAKAUER, K., VENTURA, J., JEPSEN, J.R.M., NORDENTOFT, M., 2017. Examining speed of processing of facial emotion recognition in individuals at ultra-high risk for psychosis: associations with symptoms and cognition. *Schizophrenia Research,* 185, 562-563.

GRANO, N., KARJALAINEN, M., RANTA, K., LINDGREN, M., ROINE, M. & THERMAN, S. 2016. Community-oriented family-based intervention superior to standard treatment in improving depression, hopelessness and functioning among adolescents with any psychosis-risk symptoms. *Psychiatry Research,* 237**,** 9-16.

HOLZER, L., URBEN, S., PASSINI, C. M., JAUGEY, L., HERZOG, M. H., HALFON, O. & PIHET, S. 2014. A Randomized Controlled Trial of the Effectiveness of Computer-Assisted Cognitive Remediation (CACR) in Adolescents with Psychosis or at High Risk of Psychosis. *Behavioural and Cognitive Psychotherapy,* 42**,** 421-434.

JANSSEN, H., MAAT, A., SLOT, M. I. E. & SCHEEPERS, F. 2021. Efficacy of psychological interventions in young individuals at ultra-high risk for psychosis: A naturalistic study. *Early Intervention in Psychiatry,* 15**,** 1019-1027.

KAY, S. R., FISZBEIN, A., OPLER, L. A. 1987. The Positive and Negative Syndrome Scale (PANSS) for schizophrenia. *Schizophrenia Bulletin*. 13(2): 261–276

MCAUSLAND, L. & ADDINGTON, J. 2018. Biofeedback to treat anxiety in young people at clinical high risk for developing psychosis. *Early Intervention in Psychiatry,* 12**,** 694-701.

MCFARLANE, W. R., LEVIN, B., TRAVIS, L., LUCAS, F. L., LYNCH, S., VERDI, M., WILLIAMS, D., ADELSHEIM, S., CALKINS, R., CARTER, C. S., CORNBLATT, B., TAYLOR, S. F., AUTHER, A. M., MCFARLAND, B., MELTON, R., MIGLIORATI, M., NIENDAM, T., RAGLAND, J. D., SALE, T., SALVADOR, M. & SPRING, E. 2015. Clinical and Functional Outcomes After 2 Years in the Early Detection and Intervention for the Prevention of Psychosis Multisite Effectiveness Trial. *Schizophrenia Bulletin,* 41**,** 30-43.

MEI, C., VAN DER GAAG, M., NELSON, B., SMIT, F., YUEN, H. P., BERGER, M., KRCMAR, M., FRENCH, P., AMMINGER, G. P., BECHDOLF, A., CUIJPERS, P., YUNG, A. R. & MCGORRY, P. D. 2021. Preventive interventions for individuals at ultra high risk for psychosis: An updated and extended meta-analysis. *Clinical Psychology Review,* 86.

MIKLOWITZ, D. J., O'BRIEN, M. P., SCHLOSSER, D. A., ADDINGTON, J., CANDAN, K. A., MARSHALL, C., DOMINGUES, I., WALSH, B. C., ZINBERG, J. L., DE SILVA, S. D., FRIEDMAN-YAKOOBIAN, M. & CANNON, T. D. 2014. Family-Focused Treatment for Adolescents and Young Adults at High Risk for Psychosis: Results of a Randomized Trial. *Journal of the American Academy of Child and Adolescent Psychiatry,* 53**,** 848-858.

MILLER, T. J., MCGLASHAN, T. H., WOODS, S. W., STEIN, K., DRIESEN, N., CORCORAN, C. M., HOFFMAN, R., & DAVIDSON, L. 1999. Structured Interview for Prodromal Symptoms (SIPS). *APA PsycTests*.

MOSSAHEB, N., SCHAFER, M. R., SCHLOGELHOFER, M., KLIER, C. M., COTTON, S. M., MCGORRY, P. D. & AMMINGER, G. P. 2013. Effect of omega-3 fatty acids for indicated prevention of young patients at risk for psychosis: When do they begin to be effective? *Schizophrenia Research,* 148**,** 163-167.

O'BBRIEN CANNON, A. C. O., CAPORINO, N. E., O'BRIEN, M. P., MIKLOWITZ, D. J., ADDINGTON, J. M. & CANNON, T. D. 2022. Family communication and the efficacy of family focused therapy in individuals at clinical high risk for psychosis with comorbid anxiety. *Early Intervention in Psychiatry*.

O'BRIEN, M. P., MIKLOWITZ, D. J., CANDAN, K. A., MARSHALL, C., DOMINGUES, I., WALSH, B. C., ZINBERG, J. L., DE SILVA, S. D., WOODBERRY, K. A. & CANNON, T. D. 2014. A Randomized Trial of Family Focused Therapy With Populations at Clinical High Risk for Psychosis: Effects on Interactional Behavior. *Journal of Consulting and Clinical Psychology,* 82**,** 90-101.

O'BRIEN, M. P., ZINBERG, J. L., BEARDEN, C. E., DALEY, M., NIENDAM, T. A., KOPELOWICZ, A. & CANNON, T. D. 2007. Psychoeducational multi-family group treatment with adolescents at high risk for developing psychosis. *Early Intervention in Psychiatry,* 1**,** 325-332.

OVERALL, J. E. & GORHAM, D. R. (1962).The Brief Psychiatric Rating Scale. *Psychological Reports*, 10, 790-812.

PITZIANTI, M., CASARELLI, L., PONTILLO, M., VICARI, S., ARMANDO, M. & PASINI, A. 2019. Antipsychotics Do Not Influence Neurological Soft Signs in Children and Adolescents at Ultra-High Risk for Psychosis: A Pilot Study. *Journal of Psychiatric Practice,* 25**,** 186-191.

RAUSCH, F., EIFLER, S. ESSER, A., ESSLINGER, C., SCHIRMBECK, F., MEYER-LINDENBERG, A., ZINK, M. 2013. The Early Recognition Inventory ERIraos detects at risk mental states of psychosis with high sensitivity. *Comprehensive Psychiatry*, 54(7),1068-1076.

RIECHER-RÖSSLER, A., ASTON, J., VENTURA, J., MERLO, M., BORGWARDT, S., GSCHWANDTNER, U., STIEGLITZ, R. (2008). The Basel Screening Instrument for Psychosis (BSIP): Development, structure, reliability and validity. *Fortschritte der Neurologie-Psychiatrie*. 76. 207-16. 10.1055/s-2008-1038155.

SALAZAR DE PABLO, G., GUINART, D., CORNBLATT, B., AUTHER, A.,CARRION, R., CARBON, M., JIMÉNEZ-FERNANDEZ, S.,VERNAL, D., WALITZA, S., GERSTENBERG, M., SABA, R., LO C. N., BRANDIZZI, M., ARANGO, C., MORENO, C., VAN METER, A. & FUSAR-POLI, P. & CORRELL, C. (2020). DSM-5 Attenuated Psychosis Syndrome in Adolescents Hospitalized With Non-psychotic Psychiatric Disorders. *Frontiers in Psychiatry*. 11.

STAIN, H. J., BUCCI, S., BAKER, A. L., CARR, V., EMSLEY, R., HALPIN, S., LEWIN, T., SCHALL, U., CLARKE, V., CRITTENDEN, K. & STARTUP, M. 2016. A randomised controlled trial of cognitive behaviour therapy versus non-directive reflective listening for young people at ultra high risk of developing psychosis: The detection and evaluation of psychological therapy (DEPTh) trial. *Schizophrenia Research,* 176**,** 212-219.

URBEN, S., PIHET, S., JAUGEY, L., HALFON, O. & HOLZER, L. 2012. Computer-assisted cognitive remediation in adolescents with psychosis or at risk for psychosis: a 6-month follow-up. *Acta Neuropsychiatrica,* 24**,** 328-335.

VOLLMER-LARSEN, A., HANDEST, P., PARNAS, J. 2007. Reliability of measuring anomalous experience: the Bonn Scale for the Assessment of Basic Symptoms. *Psychopathology.* 40(5):345-8.

WAITE, F., ČERNIS, E., KABIR, T., IREDALE, E., JOHNS, L., MAUGHAN, D., DIAMOND, R., SEDDON, R., WILLIAMS, N., YU, L. M., FREEMAN, D. 2023. A targeted psychological treatment for sleep problems in young people at ultra-high risk of psychosis in England (SleepWell): a parallel group, single-blind, randomised controlled feasibility trial. Lancet Psychiatry. (9):706-718. doi: 10.1016/S2215-0366(23)00203-1.

WOODBERRY, K. A., CHOKRAN, C., JOHNSON, K. A., NUECHTERLEIN, K. H., MIKLOWITZ, D. J., FARAONE, S. V. & SEIDMAN, L. J. 2021. Computer-aided learning for managing stress: A feasibility trial with clinical high risk adolescents and young adults. *Early Intervention in Psychiatry,* 15**,** 471-479.

WOODS, S. W., TULLY, E. M., WALSH, B. C., HAWKINS, K. A., CALLAHAN, J. L., COHEN, S. J., MATHALON, D. H., MILLER, T. J. & MCGLASHAN, T. H. 2007. Aripiprazole in the treatment of the psychosis prodrome. *British Journal of Psychiatry,* 191**,** S96-S101.

WOODS, S. W., WALSH, B. C., HAWKINS, K. A., MILER, T. J., SAKSA, J. R., D'SOUZA, D. C., PEARLSON, G. D., JAVITT, D. C., MCGLASHAN, T. H. & KRYSTAL, J. H. 2013. Glycine treatment of the risk syndrome for psychosis: Report of two pilot studies. *European Neuropsychopharmacology,* 23**,** 931-940.

YUNG, A.R., YUEN, H.P., MCGORRY, P.D., PHILLIPS, L.J., KELLY, D., DELL'OLIO, M., FRANCEY, S.M., COSGRAVE, E.M., KILLACKEY, E., STANFORD, C., GODFREY, K., BUCKBY, J. 2005. Mapping the onset of psychosis: the Comprehensive Assessment of At-Risk Mental States. *Australia and New Zealand Journal of Psychiatry*, 39, 964–971.
